# Ionotropic Receptor-dependent cool cells control the transition of temperature preference in *Drosophila* larvae

**DOI:** 10.1371/journal.pgen.1009499

**Published:** 2021-04-07

**Authors:** Jordan J. Tyrrell, Jackson T. Wilbourne, Alisa A. Omelchenko, Jin Yoon, Lina Ni

**Affiliations:** School of Neuroscience, Virginia Tech, Blacksburg, Virginia, United States of America; National Centre for Biological Sciences, TIFR, INDIA

## Abstract

Temperature sensation guides animals to avoid temperature extremes and to seek their optimal temperatures. The larval stage of *Drosophila* development has a dramatic effect on temperature preference. While early-stage *Drosophila* larvae pursue a warm temperature, late-stage larvae seek a significantly lower temperature. Previous studies suggest that this transition depends on multiple rhodopsins at the late larval stage. Here, we show that early-stage larvae, in which dorsal organ cool cells (DOCCs) are functionally blocked, exhibit similar cool preference to that of *wild type* late-stage larvae. The molecular thermoreceptors in DOCCs are formed by three members of the Ionotropic Receptor (IR) family, IR21a, IR93a, and IR25a. Early-stage larvae of each *Ir* mutant pursue a cool temperature, similar to that of *wild type* late-stage larvae. At the late larval stage, DOCCs express decreased IR proteins and exhibit reduced cool responses. Importantly, late-stage larvae that overexpress IR21a, IR93a, and IR25a in DOCCs exhibit similar warm preference to that of *wild type* early-stage larvae. These data suggest that IR21a, IR93a, and IR25a in DOCCs navigate early-stage larvae to avoid cool temperatures and the reduction of these IR proteins in DOCCs results in animals remaining in cool regions during the late larval stage. Together with previous studies, we conclude that multiple temperature-sensing systems are regulated for the transition of temperature preference in fruit fly larvae.

## Author summary

Animals depend on their temperature-sensing systems to avoid noxious temperature extremes and to seek optimal temperatures to survive, mate, and reproduce. Some animals pursue different optimal temperatures during development. We use fruit flies as a model to investigate how temperature-sensing systems are modulated to guide animals to distinct optimal temperatures during development. While early-stage fruit fly larvae pursue a warm temperature, late-stage larvae seek a lower temperature. Previous studies find that this transition depends on multiple rhodopsin molecules. In this study, we find an additional mechanism that also contributes to this transition. At the early larval stage, a set of cool-sensing cells express a high level of cool responsive molecules, respond strongly to low temperatures, and drive animals to avoid cool regions. At the late larval stage, these cool-sensing cells become less sensitive to low temperatures due to the decreased expression of cool responsive molecules and, thus, animals remain in cool regions. Together with previous studies, we conclude that multiple temperature-sensing systems are regulated for the transition of temperature preference in fruit fly larvae.

## Introduction

Temperature sensation is vital for animals to avoid extreme temperatures and to seek optimal temperatures to survive, mate, and reproduce. Temperature sensation is particularly essential for small animals, such as fruit flies, whose body temperatures vary with ambient temperatures [1]. Many disease vectors, including mosquitoes, respond to the temperature of their warm-blooded hosts and use it to guide their blood-feeding, through which they can transmit human diseases [2–7]. Therefore, it is crucial to understand the molecular and cellular mechanisms of temperature sensation, which may provide molecular targets to prevent host-seeking behaviors in disease vectors.

Temperature sensation is distinctively regulated through developmental stages, at least in *Drosophila* larvae. While early-stage larvae pursue a warm temperature of 24°C, the preferred temperature drops significantly in the late third instar when they stop foraging and prepare for metamorphosis [8,9]. *Drosophila* possesses multiple temperature sensing pathways and their combined effects determine the thermal preference [10,11]. Previous studies show that this transition depends on multiple rhodopsins (including Rh5 and Rh6), the phospholipase C (PLC) signaling pathway (including G_q_ and PLC), and the transient receptor potential channel TRPA1 [9]. These genes are expressed at the late third instar and are required to select low temperatures. Mutants of these genes pursue a similar temperature of 24°C at both early and late third instar and do not exhibit the transition of temperature preference observed in *wild type* [9]. Here, we focus on low-temperature sensing systems and investigate whether and how low-temperature sensing pathways contribute to the transition of temperature preference in *Drosophila* larvae.

Several low-temperature sensing pathways have been identified in *Drosophila* larvae. Calcium responsiveness indicates that neurons in terminal organ ganglions (TOGs) respond to 10°C at the third instar [12], although their functional importance in cool avoidance is controversial [8,12,13]. Class III multidendritic (MD III) neurons in the body wall respond to 6°C and mediate the cold-evoked full-body contraction at the mid-third instar [14]. The TRP channels, PDK2, NOMPC, and TRPM, are required in MD III neurons for cold nociception [14]. Another class of body wall neurons, chordotonal neurons, function in temperature discrimination in the cool range (14–17.5°C) [15]. A TRPV channel, IAV, is expressed in chordotonal neurons and contributes to thermal avoidance at cool temperatures during the third instar [15]. TRPL, a TRPC channel, is also involved in avoidance to cool temperatures at the first (15–21°C) and third (14–17.5°C) instars [15,16]. Dorsal organ cool cells (DOCCs) are three cool-responsive neurons in each dorsal organ ganglion (DOG) [13]. They possess large membrane-rich “dendrite bulbs” and are required to avoid cool temperatures during the first and early second instar [13]. Three members of the Ionotropic Receptor (IR) family, IR21a, IR93a, and IR25a, form cool receptors that control the cool responsiveness of DOCCs and drive thermotactic behavior [17–19]. Ectopic expression of IR21a in adult heating cells (HCs) confers cool sensitivity upon HCs [17,19,20]. This cool sensitivity depends on the endogenous IR93a and IR25a [17]. Thus, IR21a, IR93a, and IR25a are three subunits of the cool receptors in DOCCs. These IRs also specify the morphogenesis of the “dendrite bulbs” in adult cooling cells [19]. However, no study has determined the function of low-temperature sensing pathways in the transition of temperature preference between early and late third-instar larvae.

In this study, we investigate the role of cool-sensory pathways in the transition of temperature preference in *Drosophila* larvae. *Drosophila* larvae pursue a warm temperature (24°C) at early larval stages, including the second, early, and mid-third instar, but a cooler temperature (18–20°C) at the late third instar. DOCCs and their cool molecular receptors, formed by IR21a, IR93a, and IR25a, are indispensable for cool avoidance at early larval stages. Early-stage larvae, in which DOCCs are functionally blocked, or *Ir21a*, *Ir93a* or *Ir25a* is mutated, exhibit a preference for 18–20°C. Late third-instar larvae express decreased IR proteins and their DOCCs are less sensitive to cool temperatures. Importantly, overexpression of IR21a, IR93a, and IR25a in DOCCs directs animals to 24°C at the late third instar. Therefore, IR21a, IR93a, and IR25a in DOCCs navigate early-stage larvae to avoid 18–20°C and pursue 24°C, while reduction of these IR proteins in DOCCs causes animals to remain at 18–20°C during the late larval stage. Taken together, our findings identify a cool sensing pathway that is critical for the transition of temperature preference in *Drosophila* larvae.

## Results

### *Drosophila* larvae seek a lower temperature during the late third instar

**Fig 1:**
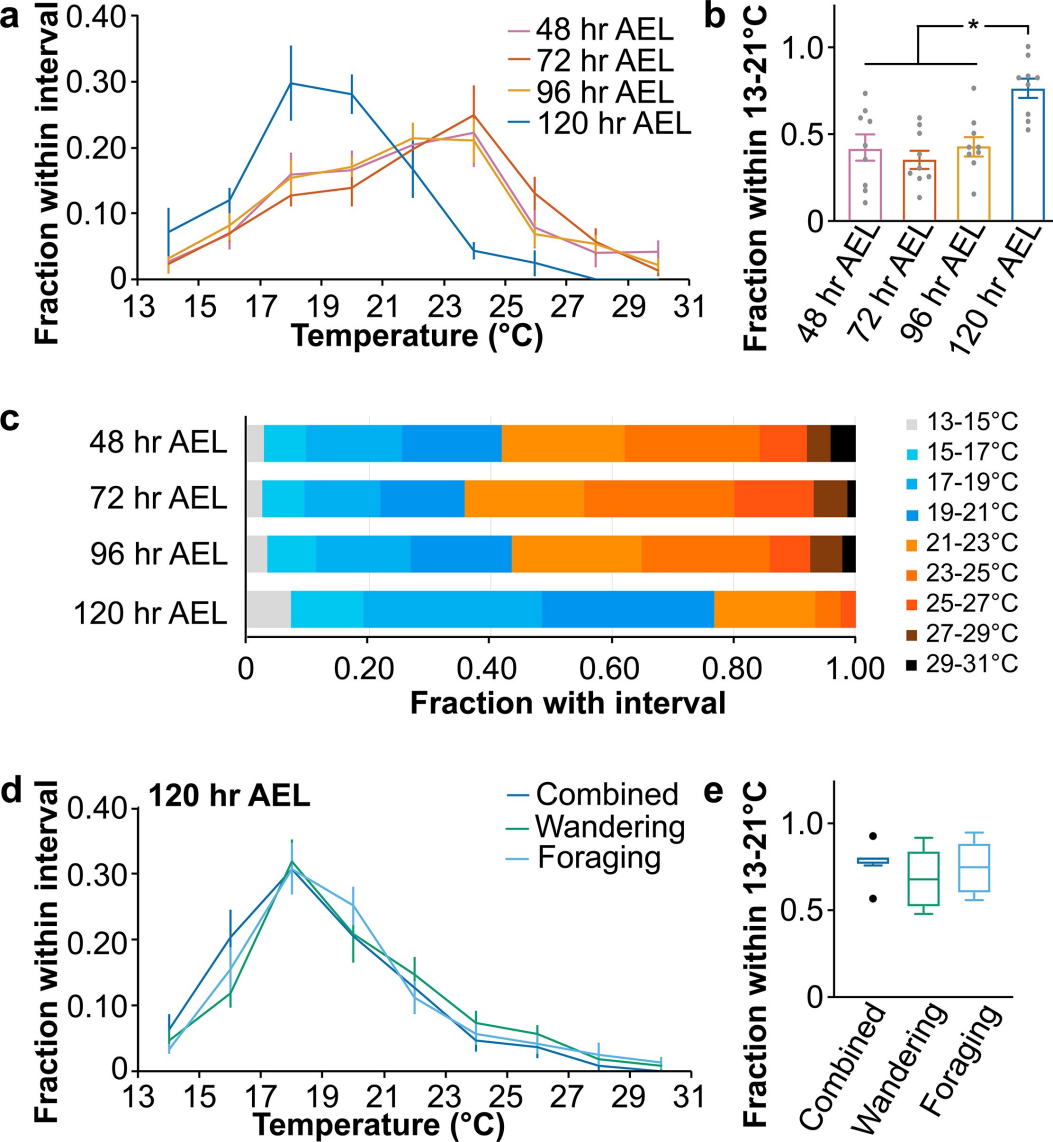
The switch of temperature preference in *Drosophila* larvae. Fig 1A. Distribution of 48 hr, 72 hr, 96 hr, and 120 hr AEL *wild type* larvae along a thermal gradient. Data represent mean ± s.e.m; n = 9. Fig 1B. Fraction of larvae of indicated ages in the 13–21°C region. Scatterplots are superimposed with bars that represent mean ± s.e.m. Ordinary one-way ANOVA, F = 9.31; * *p* < 0.01, Tukey HSD. Fig 1C. Mean percentages of 48 hr, 72 hr, 96 hr, and 120 hr AEL *wild type* larvae in each temperature zone from 13°C to 31°C. Fig 1D. Distribution of foraging- and wandering-stage larvae along a thermal gradient. Combined: larvae at 120 hr AEL, including both wandering- and foraging-stage larvae. Data represent mean ± s.e.m; n = 9. Fig 1E. Fraction of larvae of indicated ages in the 13–21°C region. Boxes are defined by 25th to 75th percentiles; internal lines show median; whiskers extend 1.5 times interquartile range; black dots denote outliers. Kruskal-Wallis test, *p* = 0.5206.

To understand *Drosophila* larval thermotactic behaviors, we set up a temperature gradient from 13–31°C (**S1 Fig**). *Drosophila* larvae pursued 24°C during the early stages, including the second instar (48 hr After Egg Laying (AEL)), early third instar (72 hr AEL), and mid-third instar (96 hr AEL) (**Fig 1A and 1C**). First-instar larvae (24 hr AEL) had limited mobility and were not examined. However, *Drosophila* larvae preferred a lower temperature of 18–20°C during the late third instar (120 hr AEL) (**Fig 1A and 1C**). To quantify the thermal preference, we calculated the fraction of larvae within the 13–21°C region (21°C is halfway between 18°C and 24°C) and found that a significantly higher fraction of late third-instar larvae chose the 13–21°C region than early-stage larvae (**Fig 1B**). This observation is consistent with a previous report that the late third-instar larvae pursued a lower temperature [9]. In the following study, we used early (72 hr AEL) and late third-instar (120 hr AEL) larvae to understand the mechanism for the transition of thermal preference in *Drosophila* larvae.

At 120 hr AEL, *Drosophila* larvae started to cessate foraging and transited to the wandering stage. To examine whether larvae exhibited a distinct temperature preference in the foraging and wandering stages, we analyzed their thermotactic behavior and did not detect a difference in thermal preference during both stages (**Fig 1D and 1E**).

### The role of DOCCs in cool avoidance

**Fig 2:**
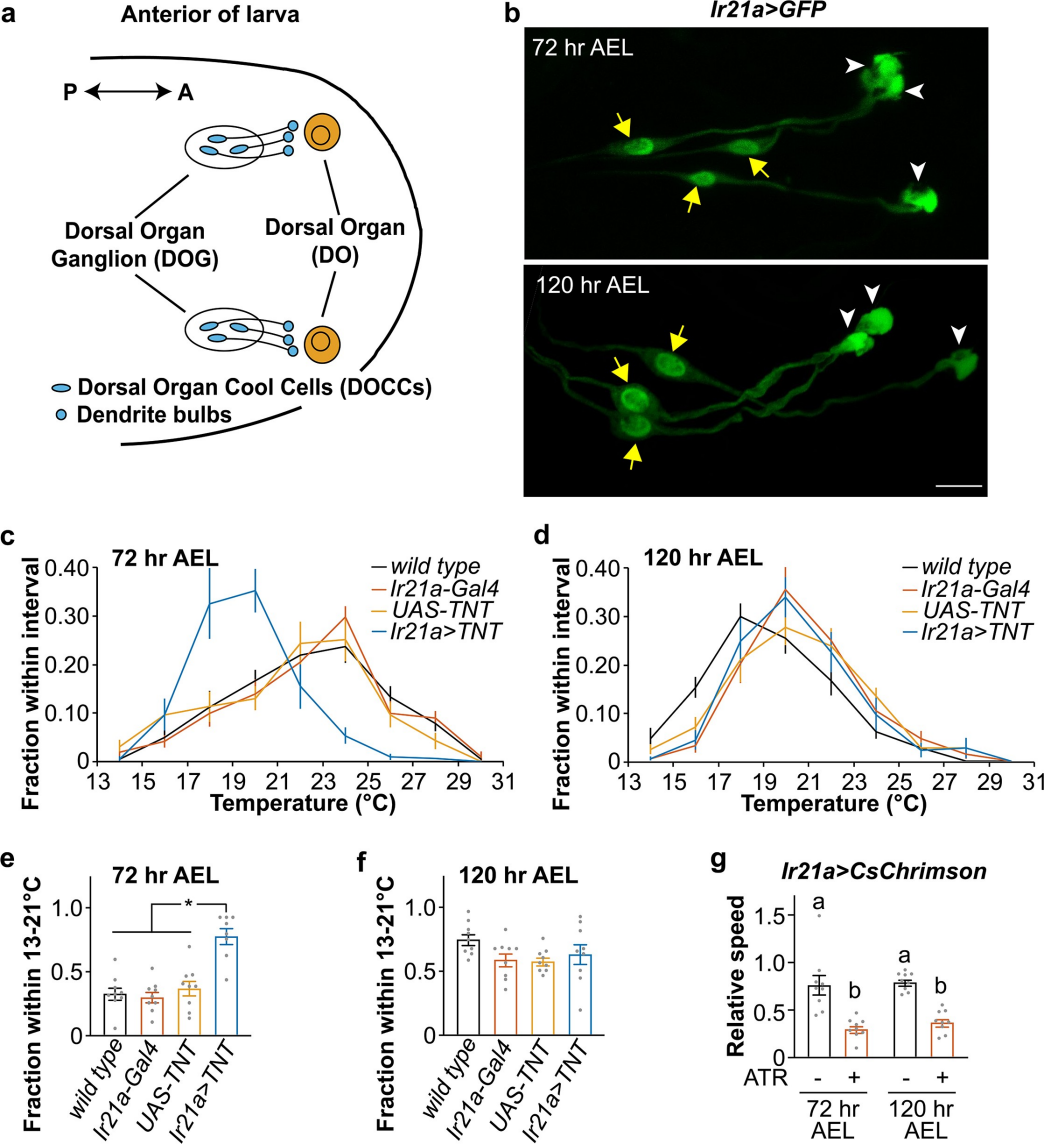
The role of DOCCs in the transition of temperature preference. Fig 2A. Third-instar larval anterior. Each dorsal organ ganglion (DOG) contains three DOCCs (blue ovals). Each DOCC possesses a “dendrite bulb” (blue circles). The double-headed arrow denotes the anterior-posterior axis. Fig 2B. *Ir21a-GAL4;UAS-GFP* (*Ir21a>GFP*) labels DOCCs at 72 hr (top) and 120 hr (bottom) AEL. Yellow arrows denote cell bodies and white arrowheads denote “dendrite bulbs.” Scale bars, 10 μm. Fig 2C and 2D. Larvae distribution along a thermal gradient of indicated genotypes and ages. Data represent mean ± s.e.m; n = 9, except n = 8 for *Ir21a>TNT* (*Ir21a-Gal4/UAS-TNT*) at 72 hr AEL. Fig 2E and 2F. Fraction of 72 hr (Fig 2E) and 120 hr (Fig 2F) AEL larvae of indicated genotypes in the 13–21°C region. Ordinary one-way ANOVA; (Fig 2E) F = 17.56; * *p* < 0.0001, Tukey HSD; (Fig 2F) F = 2.165, *p* = 0.1115. Fig 2G. Relative speed of 72 hr and 120 hr AEL larvae when DOCCs express *CsChrimson* with or without dietary retinal (ATR). Relative speed is defined as the moving speed during red light on divided by the moving speed during light off. The genotype of *Ir21a>CsChrimson* is *Ir21a-Gal4;UAS-CsChrimson*. n = 9. Ordinary one-way ANOVA, F = 18.42; letters denote statistically distinct groups, *p* < 0.001, Tukey HSD.

At the first instar, DOCCs control cool avoidance and are specifically labeled by *Ir21a-Gal4* [17]. At 72 hr AEL and 120 hr AEL, *Ir21a-Gal4* was expressed in three neurons in each DOG (**Fig 2A and 2B**). Within these neurons, robust GFP signals were observed in the cell bodies (yellow arrows) and “dendrite bulbs” (white arrowheads), indicating that they are DOCCs. DOCC cell bodies and their “dendrite bulbs” are intact at the late third instar and morphological defects are not observed.

To address the function of DOCCs in cool avoidance at the early and late third instar, we blocked the function of DOCCs by expressing the synaptic neurotransmitter blocker, tetanus toxin light chain (TNT) [21] using *Ir21a-Gal4*. At 72 hr AEL, these larvae concentrated in the 18–20°C region (**Fig 2C and 2E**), suggesting that DOCCs are required to avoid 18–20°C during the early third instar. In contrast, blockage of DOCCs did not affect the temperature preference at 120 hr AEL (**Fig 2D and 2F**), indicating that DOCCs are dispensable for the temperature preference at the late third instar.

At 72 hr AEL, functional blockage of DOCCs shifted the temperature preference to 18–20°C, similar to the temperature preference of *wild type* larvae at 120 hr AEL (**S2 Fig**). These data suggest that the preference of late third-instar larvae to 18–20°C may be due to the downregulation of the activity of DOCCs or the DOCC-dependent neural pathway at 120 hr AEL.

To investigate whether the neural circuit downstream of DOCCs is inactivated at the late third instar, optogenetics was applied. DOCCs drive cool avoidance behavior [13]. We used the red light-shifted channelrhodopsin CsChrimson to activate DOCCs [22]. At 72 hr AEL, red light-mediated DOCC activation drove aversive behaviors, including the pause of run, which in turn led to the decrease of the run speed–a parameter that negatively correlates with aversive behaviors (**Figs 2G and S3 and S1 Movie**) [23,24]. These aversive behaviors reflect the cool avoidance driven by DOCCs on a thermal gradient. At 120 hr AEL, DOCC activation drove similar behaviors (**Figs 2G and S3 and S1 Movie**). Not only DOCCs but also the neural circuit downstream of DOCCs is necessary for the avoidance behavior. Since CsChrimson is expressed in DOCCs, red light activates DOCCs, which in turn activates the downstream neural circuit. If the downstream neural circuit is inactivatable, activation of DOCCs by red light would not cause the aversive behaviors. Therefore, the optogenetic data suggest that, at the late third instar, the DOCC-dependent neural pathway is activatable and can drive the avoidance behavior. Thus, we propose that the thermal responses of DOCCs, not their downstream neural circuit, are downregulated at the late third instar.

### DOCCs exhibit reduced cool responses at the late third instar

**Fig 3:**
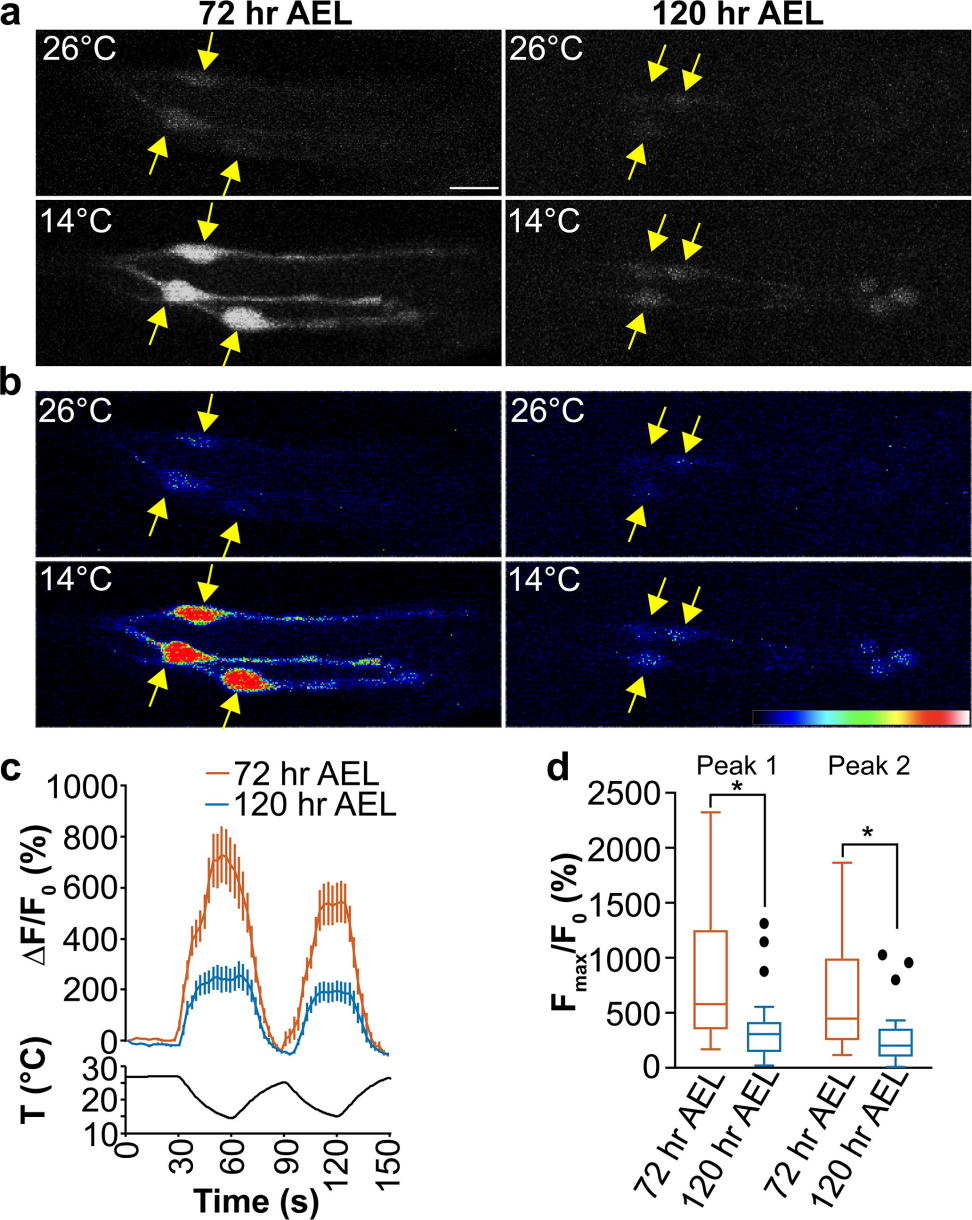
DOCCs exhibit lower cool responses at the late third instar. Fig 3A and 3B. Temperature responses of *Ir21a-Gal4;UAS-GCaMP6m*-labeled DOCCs at 72 hr (left) and 120 hr (right) AEL. (Fig 3A) raw images; (Fig 3B) colors reflect fluorescence intensity. Yellow arrows denote cell bodies. Scale bars, 10 μm. Fig 3C. Fluorescence is quantified as the percent change in fluorescence intensity compared to initial intensity. 72 hr AEL, n = 33 cells from 10 animals; 120 hr AEL, n = 30 cells from 11 animals. Traces, mean ± s.e.m. Fig 3D. Ratio of maximum fluorescence versus initial fluorescence during the first and second cooling phase at 72 hr and 120 hr AEL. Mann-Whitney test, * *p* < 0.001.

To assess the physiological responses of DOCCs to cool, we expressed a genetically encoded calcium indicator GCaMP6m [25] with *Ir21a-Gal4*. At 72 hr AEL, when exposed to a sinusoidal temperature stimulus between ~26°C and ~14°C, GCaMP6m fluorescence in DOCCs increased upon cooling and decreased upon warming (**Fig 3 and S2 Movie**). The fluorescence in DOCCs only subtly declined when samples were held at constant cool temperatures for 60 seconds, but rapidly dropped to the pre-stimulus level upon warming (**S4 Fig**). At 120 hr AEL, DOCCs exhibited significantly reduced responses to cooling when exposed to similar temperature stimuli (**Fig 3 and S2 Movie**). These data suggest that the cool responses of DOCCs are significantly reduced during the late third instar. Since DOCCs drive an avoidance behavior, the strong responses of DOCCs to cool temperatures navigate early third-instar larvae to avoid cool regions. When the cool responses reduce, this avoidance decreases.

### DOCCs express fewer cool receptors during the late third instar

**Fig 4:**
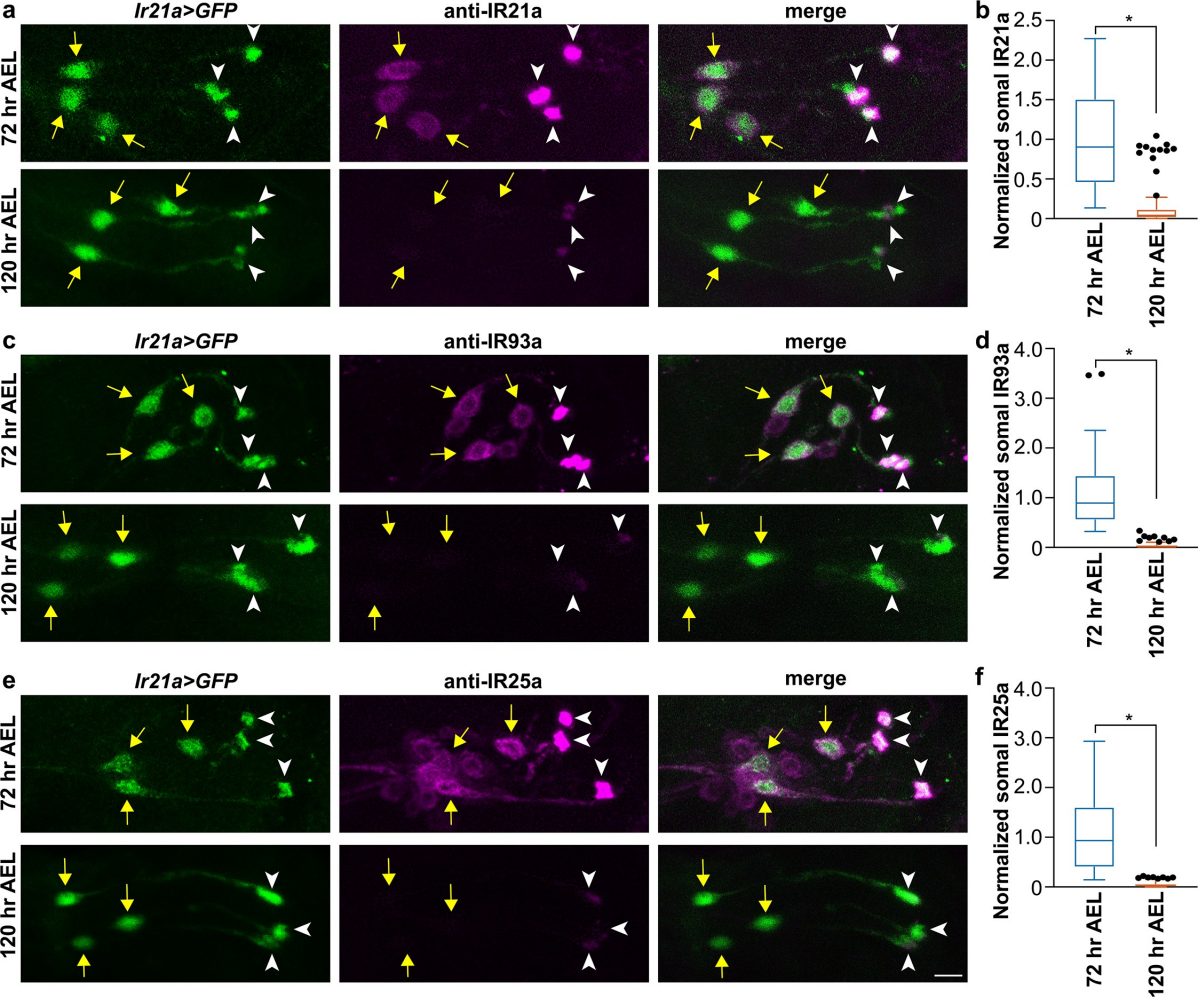
Expression of IR21a, IR93a, and IR25a proteins decreases in DOCCs at the late third instar. Fig 4A, 4C, and 4E. IR21a (Fig 4A), IR93a (Fig 4C), and IR25a (Fig 4E) immunostaining. Left: *Ir21a>GFP-*labeled DOCCs. Middle: Expression of IR21a (Fig 4A), IR93a (Fig 4C), and IR25a (Fig 4E) proteins in the dorsal organ. Right: *Ir21a>GFP-*labeled DOCCs express IR21a (Fig 4A), IR93a (Fig 4C) and IR25a (Fig 4E) proteins at 72 hr AEL (top), which are significantly decreased at 120 hr AEL (bottom). Yellow arrows denote cell bodies and white arrowheads denote “dendrite bulbs.” The genotype of *Ir21a>GFP* is *Ir21a-Gal4;UAS-GFP*. Scale bars, 10 μm. Fig 4B, 4D, and 4F. Normalized somal fluorescent intensity of IR21a (Fig 4B), IR93a (Fig 4D) and IR25a (Fig 4F) at 72 hr AEL and 120 hr AEL. Mann-Whitney test, * *p* < 0.001. IR21a: n = 84 cells from 14 animals at 72 hr AEL and n = 109 cells from 21 animals at 120 hr AEL. IR93a: n = 69 cells from 12 animals at 72 hr AEL and n = 90 cells from 16 animals at 120 hr AEL. IR25a: n = 76 cells from 13 animals at 72 hr AEL and n = 56 cells from 12 animals at 120 hr AEL.

We then asked whether reduced cool responses in DOCCs were due to a lower expression of IR21a, IR93a, and IR25a, which form the cool receptors in DOCCs in first-instar larvae [17,18]. We examined the expression of each IR in DOCCs at 72 hr AEL and 120 hr AEL by staining larvae with antisera for IR21a, IR93a, and IR25a [18,19,26] (**Fig 4**). At 72 hr AEL, robust IR21a protein (magenta) was specifically detected in three *Ir21a-Gal4*-expressing DOCCs (green) (**Fig 4A**). IR93a protein (magenta) was expressed in five neurons, including three *Ir21a-Gal4*-expressing DOCCs (green) (**Fig 4C**). IR25a protein (magenta) was detected in multiple cells, as well as three *Ir21a-Gal4*-expressing DOCCs (green) (**Fig 4E**). Robust IR proteins were detected in “dendrite bulbs,” which is consistent with a role of these IRs in thermosensing (**Fig 4A, 4C and 4E;** white arrowheads). At 120 hr AEL, expression of each IR protein in DOCCs was significantly decreased (**Fig 4**). In adults, lack of IR21a, IR93a, or IR25a results in degeneration of the “dendrite bulbs” in cooling cells, which can be observed by optical imaging techniques [19]. Although expression of IR21a, IR93a, and IR25a was significantly decreased at the late third instar, no change in morphology of “dendrite bulbs” was observed (**Fig 4A, 4C and 4E)**. These data suggest that DOCCs express a lower level of IR21a, IR93a, and IR25a in the late third instar, which results in a decrease of cool responses in DOCCs.

### The role of IR21a, IR93a, and IR25a in cool avoidance

**Fig 5:**
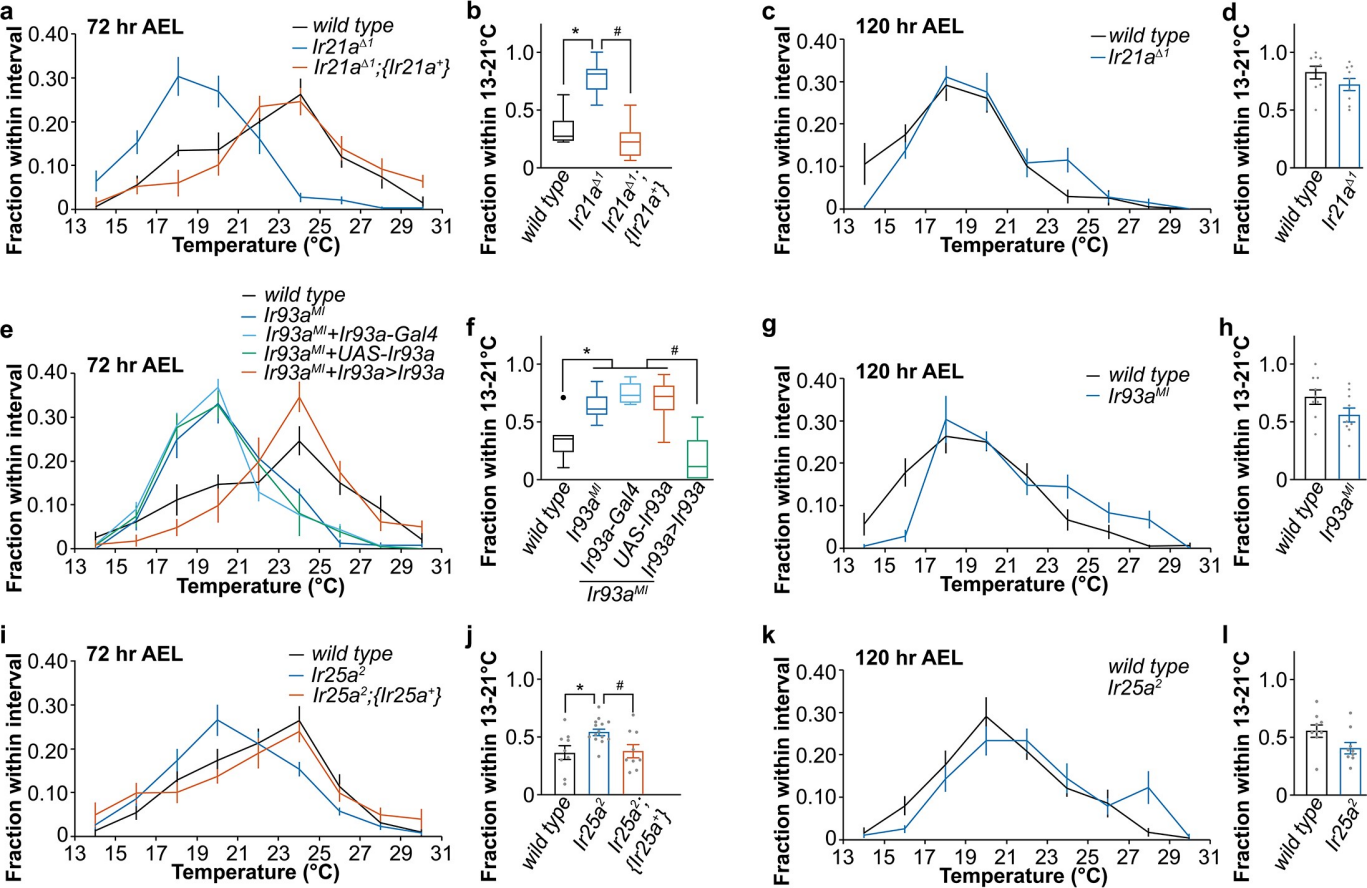
The role of IR21a, IR93a, and IR25a in the transition of temperature preference. Fig 5A, 5C, 5E, 5G, 5I, and 5K. Larvae distribution along a thermal gradient of indicated genotypes and ages. Data represent mean ± s.e.m; n = 9, except n = 14 for *Ir25a*^*2*^ at 72 hr AEL. Fig 5B, 5D, 5F, 5H, 5J, and 5L. Fraction of larvae of indicated genotypes and ages in the 13–21°C region. (Fig 5B) Mann-Whitney test. * *p* < 0.0001, comparing to *wild type*. # *p* < 0.0001, comparing to *Ir21a*^*Δ1*^*;{Ir21a*^*+*^*}*. (Fig 5D) Welch’s test, F = 1.075, *p* = 0.1813. (Fig 5F) Mann-Whitney test. * *p* < 0.01, comparing to *wild type*. # *p* < 0.001, comparing to *Ir93a*^*MI*^,*Ir93a>Ir93a* (*Ir93a*^*MI*^,*Ir93a-Gal4/UAS-mCherry*:*Ir93a*). (Fig 5H) Welch’s test, F = 1.065, *p* = 0.0918. (Fig 5J) Welch’s test, *wild type* vs *Ir25a*^*2*^: F = 2.878, *wild type* vs *Ir25a*^*2*^*;{Ir25a*^*+*^*}*: F = 1.103, *Ir25a*^*2*^ vs *Ir25a*^*2*^*;{Ir25a*^*+*^*}*: F = 2.608. * *p* < 0.05, comparing to *wild type*. # *p* < 0.05, comparing to *Ir25a*^*2*^*;{Ir25a*^*+*^*}*. (Fig 5L) Welch’s test, F = 1.172, *p* = 0.0536.

To understand the role of IR21a, IR93a, and IR25a in cool avoidance, we analyzed the thermotactic behavior in *Ir21a*^*Δ1*^, *Ir93a*^*MI*^, and *Ir25a*^*2*^ mutants [17,18,26]. At 72 hr AEL, *Ir21a*^*Δ1*^ larvae pursued a lower temperature and concentrated at 18–20°C (**Fig 5A and 5B**). An *Ir21a* genomic minigene reversed the phenotype (**Fig 5A and 5B**) [17]. These data suggest that IR21a is important in determining thermal preference, and specifically contributes to aversion of cool regions during the early third instar. By contrast, *Ir21a*^*Δ1*^ larvae exhibited a similar temperature preference to *wild type* larvae at 120 hr AEL, suggesting that IR21a is dispensable for temperature preference at the late third instar (**Fig 5C and 5D**). Similar phenotypes were observed in *Ir93a*^*MI*^ and *Ir25a*^*2*^ mutants at 72 hr AEL, which were reversed by *Ir93a* and *Ir25a-*specific rescue, respectively [18,26] (**Fig 5E, 5F, 5I, and 5J**). Neither the *Ir93a*^*MI*^ nor the *Ir25a*^*2*^ mutant had defects in cool avoidance at 120 hr AEL (**Fig 5G, 5H, 5K, and 5L**). Therefore, IR21a, IR93a, and IR25a are required to avoid cool temperatures at the early but not the late third instar. The phenomena observed in *Ir21a*^*Δ1*^, *Ir93a*^*MI*^, and *Ir25a*^*2*^ mutants were not due to developmental defects, because the time to pupation was indistinguishable between the mutants and controls (**S5 Fig**). Moreover, the *Ir21a*^*Δ1*^, *Ir93a*^*MI*^, and *Ir25a*^*2*^ mutant phenotypes were not due to the general impairment in temperature discrimination or locomotion activity, because these mutants had no defects in the aversion of 30–32°C in the two-choice thermotactic behavioral assay (**S6 Fig**).

In addition, *Ir21a*^*Δ1*^, *Ir93a*^*MI*^, and *Ir25a*^*2*^ mutants at 72 hr AEL exhibited similar temperature preferences to *wild type* larvae at 120 hr AEL (**S7 Fig**). Since the expression of IR21a, IR93a, and IR25a in DOCCs is decreased during the late third instar (**Fig 4**), we hypothesize that the low-temperature preference is, at least partially, due to a decreased expression of IR-formed cool receptors in DOCCs.

**Fig 6:**
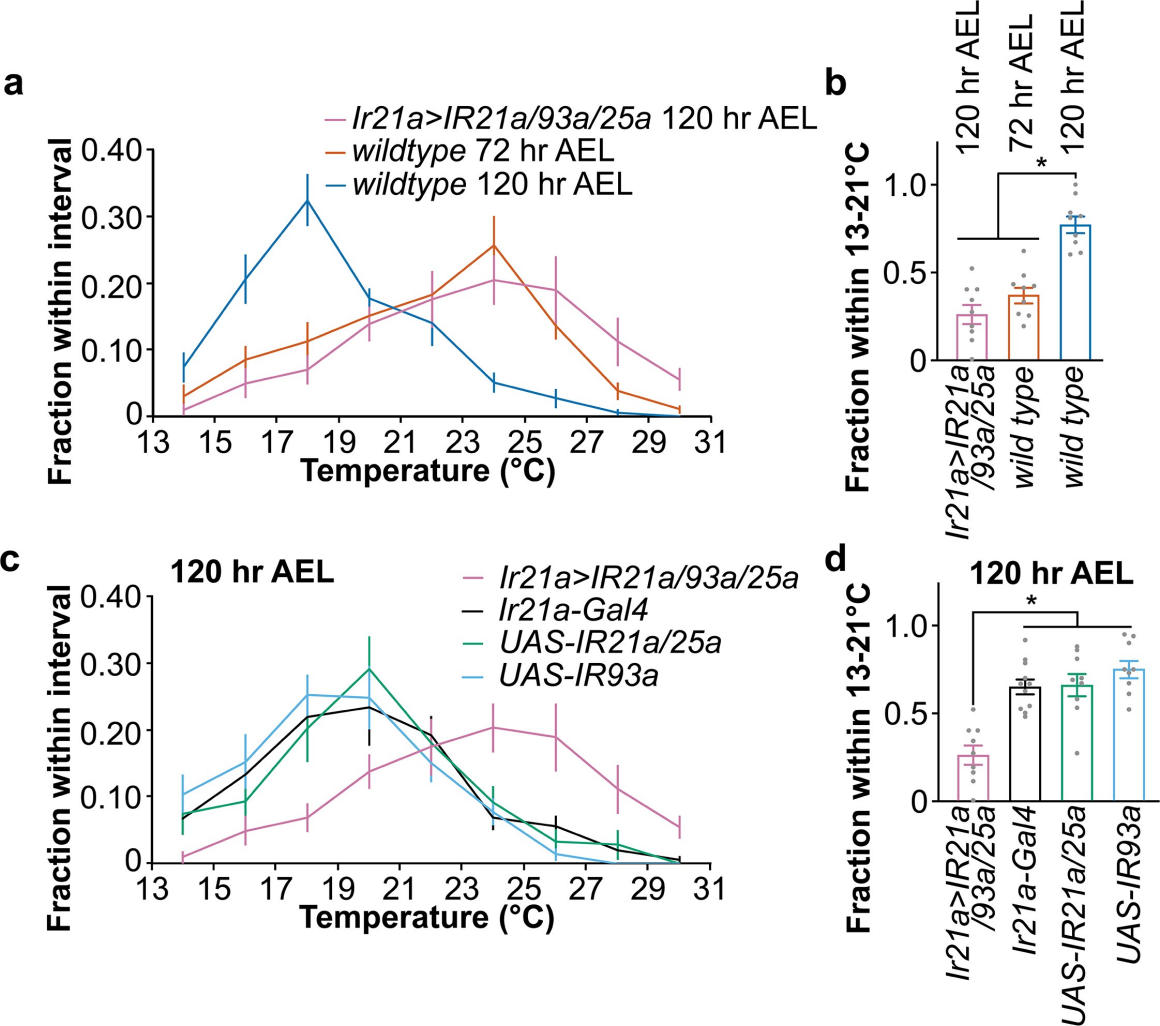
Expression of IR21a, IR93a, and IR25a in DOCCs navigates late third-instar larvae to 24°C. Fig 6A and 6C. Larvae distribution along a thermal gradient of indicated genotypes and ages. The genotype of *Ir21a>IR21a/93a/25a* is *Ir21a-Gal4/UAS-IR25a;UAS-IR21a/UAS-IR93a*. The genotype of *UAS-IR21a/25a* is *UAS-IR25a;UAS-IR21a*. Data represent mean ± s.e.m; n = 9, except n = 11 for *Ir21a-Gal4*. The same data of *Ir21a>IR21a/93a/25a* were used in **Fig 6A** and **6C**. Fig 6B and 6D. Fraction of larvae of indicated genotypes and ages in the 13–21°C region. Ordinary one-way ANOVA, F = 30.18 (Fig 6B) and F = 15.25 (Fig 6D); * *p* < 0.01, Tukey HSD.

To test this hypothesis, we expressed IR21a, IR93a, and IR25a proteins in DOCCs using *Ir21a-Gal4* (*Ir21a>IR21a/93a/25a*) and analyzed the thermotactic behavior at 120 hr AEL [17,18,27]. While controls pursued cooler temperatures at 120 hr AEL (**Fig 6**), expression of IR21a, IR93a, and IR25a proteins in DOCCs directed animals to 24°C, similar to the preferred temperature of *wild type* larvae at 72 hr AEL (**Fig 6A and 6B**). Correspondingly, expression of IR21a, IR93a, and IR25a was significantly increased at 120 hr AEL in *Ir21a>IR21a/93a/25a* compared to *wild type* (**S8 Fig**). Of note, at 72 hr AEL, other IR25 and IR93a positive cells were detected beyond three DOCCs (**Figs 4C and 4E and S8A and S8D**). In contrast, in *Ir21a>IR21a/93a/25a* animals at 120 hr AEL, these other positively staining cells were barely observed compared to three DOCCs (**S8C and S8F Fig**). These data support the hypothesis that the reduction of IRs in DOCCs causes late third-instar larvae to remain at a lower temperature.

## Discussion

In this study, we identified a mechanism underlying the transition of temperature preference in *Drosophila* larvae. *Drosophila* larvae pursue a warm temperature (24°C) during early larval stages, but a cooler temperature (18–20°C) during the late third instar. Our findings indicate that the transition of thermal preference is, at least partially, due to reduced expression of cool thermoreceptors, formed by IR21a, IR93a, and IR25a, in DOCCs at the late third instar. Reduced expression of cool receptors leads to decreased cool responses of DOCCs, which, in turn, limits the ability of late third-instar larvae to avoid cool temperatures.

*Drosophila* possesses multiple temperature sensing pathways [10,11] and thermal preference results from the combined effects of these pathways. At different developmental stages, *Drosophila* exhibits distinct thermal preference [9]. On a shallow temperature gradient, early third-instar larvae pursue 24°C and late third-instar larvae seek 18–20°C. Previous studies identified that mutants in the rhodopsin signaling pathway, including Rh5/6, G_q_, PLC (encoded by *norpA*), and TRPA1, pursue 24°C at the late third instar [9], suggesting that these genes function at the late third instar in driving animals to low temperatures. Driver lines showed that Rh5 and Rh6 are coexpressed with TRPA1 in the body wall. A recent study identified that Rh6 and a different PLC, PLC21C, are necessary for the cool activation of adult bitter neurons [28]. If the function of Rh6 in cool sensing is conserved throughout the development, then the rhodopsin pathway, at the late third instar, drives an attractive behavior. Alternatively, the rhodopsin pathway, including Rh5/6, G_q_, PLC, and TRPA1, may be required for sensing 24°C and drives an avoidance behavior.

In contrast, we focused on another thermosensing system that functions in a different developmental stage and is also critical for the transition of temperature preference in *Drosophila* larvae. DOCCs drive an avoidance behavior. Cool receptor IR proteins are expressed in DOCCs in early-stage larvae. Lack of these IRs limits the ability of early-stage larvae to avoid 18–20°C, suggesting that these proteins navigate early-stage larvae to avoid low temperatures and move towards higher temperatures. Expression of these proteins decreases at the late third instar, which renders late third-instar larvae to possess weaker low-temperature sensing systems. Hence, late third-instar larvae do not avoid low temperatures and remain at 18–20°C. Both systems are required for the transition of thermal preference in *Drosophila* larvae: the rhodopsin pathway guides late third-instar larvae to move towards 18°C, while the IR pathway navigates early-stage larvae to avoid 18°C and move towards 24°C (**S9 Fig**). Taken together, multiple temperature sensing systems are regulated through developmental stages to navigate animals to distinct optimal temperatures. Regulatory mechanisms will be explored in the future. Other low-temperature sensing systems, including the cold-responsive neurons in TOGs [8,12,13], MD III and chordotonal neurons in the body wall [14,15], and TRPL-expressing neurons [15,16] may function in driving larvae to avoid noxious temperatures below 18°C.

To investigate the optimal temperatures through *Drosophila* larval stages, we set up a temperature gradient from 13–31°C. Using this apparatus, we demonstrated that *Drosophila* larvae pursue 24°C during early larval stages and 18–20°C during the late third instar. Previous studies used a temperature gradient from 18–28°C [9,29]. In the previous study, animals accumulated in the area of lowest temperature (18°C), which does not exclude the possibility that larvae prefer a temperature that is lower than 18°C. Thus, they were unable to determine the optimal temperature during the late third instar. In contrast, our apparatus clearly shows that late third-instar larvae prefer 18–20°C.

The following two observations support the conclusion that the neural pathway downstream of DOCCs does not contribute to the transition of thermal preference. First, red light-activated DOCCs drive similar avoidance to light during the early and late third instar. Second, overexpression of IR21a, IR93a, and IR25a in DOCCs navigates animals to 24°C in the late third instar. Further identification of this neural circuit could help to clarify the function of neurons downstream of DOCCs in the transition of temperature preference.

Although IRs specify the morphogenesis of the “dendrite bulbs” in adult cooling cells [19], morphological change of DOCCs’ dendrite endings was not observed at the late third instar (**Figs 2B, 4A, 4C, and 4E**), suggesting that reduction of IRs at the late third instar has no, or mild, effects on the morphogenesis of the “dendrite bulbs” in DOCCs. The residual expression of IRs might be able to maintain the intact structure of “dendrite bulbs.” It is also possible that IR reduction occurs within a short time window (from 72 hr AEL, if not later, to 120 hr AEL) and thus the morphological change could be mild and not be detected by optical imaging techniques. Alternately, IRs might not function in the morphogenesis of the “dendrite bulbs” in DOCCs. Further studies will test these possibilities.

IRs are thought to form heterotetrameric complexes [27,30,31] and IR21a, IR93a, and IR25a are subunits of the cool receptors. However, it has not been identified whether these three IRs are sufficient to form a functional ion channel in response to cool temperatures or whether a fourth IR is necessary. Overexpression of IR21a, IR93a, and IR25a directs animals to 24°C during the late third instar, suggesting that these three IRs might be sufficient to sense cool temperatures. However, we cannot exclude the possibility that a fourth IR is necessary and its expression is not, or less, reduced during the late third instar.

Moreover, IR21a, IR93a, and IR25a form a phasic sensor in adult cooling cells [19]. Our calcium imaging showed that the DOCCs’ responses to cool temperatures barely declined when samples were held at constant cool temperatures for a minute (**S4 Fig**). This inconsistency may be due to the different temperatures that were used between these two studies or different factors in DOCCs and adult cooling cells that regulate the properties of the cool sensor formed by IR21a, IR93a, and IR25a.

In summary, our findings identify a mechanism underlying the transition of temperature preference in *Drosophila* larvae. Since adult flies also pursue 24°C, it will be interesting to understand the physiological needs of temporally pursuing 18–20°C during the late third instar. Another key question is to understand, at the late third instar, the regulatory mechanisms for the expression of IRs and other thermosensory molecules. Transcriptional and translational mechanisms of regulation will be explored.

## Materials and methods

### Fly strains

*CS* was used as the *wild type* control. The following flies were previously described: *UAS-TNT* (*UAS-TeTxLC*) [21], *Ir21a-Gal4* [17], *UAS-GFP* (*p{10XUAS-IVS-Syn21-GFP-p10}attp2*) [32], *UAS-CsChrimson* [22], *UAS-GCaMP6m* (*P{20XUAS-IVS-GCaMP6m}attp2*) [25], *Ir21a*^*Δ1*^ [17], *Ir25a*^*2*^ [26], *Ir93a*^*MI*^ [18], *{Ir25a*^*+*^*}* (*BAC{Ir25a*^*+*^*}*) [33], *{Ir21a*^*+*^*}* [17], *UAS-mCherry*:*Ir93a* [18], *UAS-Ir93a* [18], *Ir93a-Gal4* [34], *UAS-Ir21a* [17], *UAS-Ir25a* [27].

### Larvae preparation and aging

To prepare synchronized larvae for assays, flies were maintained at 25°C under 12-hour light/12-hour dark cycles. They were given at least 24 to 48 hours to recover from CO_2_ before being used to prepare larvae. Each vial contained 20 to 45 male and 20 to 45 female flies. To prepare the larvae, the flies were tapped over to new vials containing yeast granules and were allowed 2 to 8 hours to lay eggs. The beginning of this egg-laying period initiated larvae aging (early, mid, and late third-instar). Typically, 72 hr AEL coincided with the initiation of third instar, while 120 hr AEL was a mix of wandering-stage and foraging-stage larvae. During the aging period, larvae vials were given diH_2_O to moisten the environment and food as needed.

Larvae were collected at respective ages (72 hr, 96 hr, and 120 hr AEL) using 10 mL of 20% w/v sucrose solution. After 20 minutes, the larvae were collected and thoroughly cleaned three times with diH_2_O. The larvae were then plated on a 60 mm tissue culture dish (Corning) with 13 mL of 3%, room temperature (about 20°C) agar gel and given 5 to 10 minutes to recover from the washing process. To ensure that larvae were third instar and not second instar, they were examined under a dissecting microscope to confirm that the anterior spiracles were branched and posterior spiracles had an orange ring at their tip. Furthermore, immobile larvae were discarded at this stage due to initiating the prepupal stage.

To separate wandering-stage and foraging-stage larvae at 120 hr AEL (**Fig 1D and 1E**), wandering-stage larvae were first collected from the side of the vial. Then the sucrose solution was added to collect foraging-stage larvae.

### Larvae aging experiment

To prepare synchronized larvae for the aging experiment, larvae were prepared as described above. Flies were given 4–7 days to recover from CO_2_ before being used to prepare larvae. The number of pupae in each vial was counted at each of the following time intervals: 24 hr, 48 hr, 72 hr, 96 hr, 120 hr, 144 hr, 168 hr, 192 hr, 216 hr, and 240 hr AEL. Vials with less than 30 pupae were discounted due to the inaccurate analysis of a small sample size. Vials with more than 130 pupae were also discounted because crowded environments elongated the time to pupation. Since *Ir21a*^*Δ1*^ is balanced with *CyoαGFP*, only non-fluorescent animals were counted. The distribution of pupated larvae was calculated as follows: (number of pupated larvae)/(total number of pupated larvae at 240 hr AEL) x 100%.

### Larvae temperature gradient assay

The apparatus for the temperature gradient assays was an aluminum plate (61 x 30.5 x 0.6 cm) on top of an ice bath (39 x 26 x 6 cm Pyrex tray) on the right side and a hot plate (SP88850200, Thermo Scientific) on the left side set to approximately 70°C. The aluminum plate was 13.5 cm on the hot plate and 19.5 cm on the ice bath. On top of the aluminum plate was 800 mL of 3% agar gel (36 x 24 x 0.9 cm) situated in the middle of the plate. This setup generated a steady temperature gradient from 13 to 31°C. The temperature on the surface of the gel was monitored using a surface temperature probe (80PK-3A, Fluke) and thermometer (Fisherbrand Traceable Big-Digit Type K Thermometer). From 13 to 31°C, every 1°C was located on the temperature gradient and demarcation was made at that location, resulting in 18 demarcations. A 1 cm perimeter demarcation was also made on the edge of the gel. The gel was lightly sprayed with diH_2_O and covered with plastic wrap while forming the temperature gradient to prevent the gel from drying out. The temperature gradient was formed within approximately 10 minutes.

Larvae were collected and prepared for the assay as detailed above. To initiate the temperature gradient assays, between 20 to 35 larvae were placed in the middle of the gel at approximately 22°C and were given between 10 to 15 minutes to make temperature selections depending on larvae age and movement speed. All of the assays were conducted in dim light condition (<10 lux) and between 7:00 a.m. and 7:00 p.m. Larvae that moved out of the gel, were within 1 cm of the edge of the gel, or were immobile were discounted from the analysis. The 1 cm demarcation on the edge of the gel was created to exclude larvae that were not making temperature selections but were seeking to move out of the gel or seeking a place to pupate. Assays with fewer than 14 larvae after these exclusion criteria were discarded. The number of larvae in each temperature zone was counted and the distribution was calculated as follows: (number of larvae in temperature zone)/(total number of larvae) x 100%.

### Larvae two-choice thermotactic behavioral assay

The two-choice assay was performed as described with some modifications [35]. A 3% agar gel (10 x 9.5 in) was evenly placed on two aluminum plates separated by 1/16 inches (the release zone). The plates were individually temperature controlled. The surface temperature was 30–32°C on one side of the gel and 24–26°C on the other. The temperature was monitored before each trial using a surface temperature probe (80PK-3A, Fluke) and thermometer (Fisherbrand Traceable Big-Digit Type K Thermometer). A *wild type* control was run at the beginning of daily experiments. Water was gently sprayed between trials to moisten the agar surface. 15 to 30 early third-instar larvae were placed at the release zone. The experiment was conducted at dim ambient light (<10 lux). The larvae on each side were counted after 2 minutes and the preference index (PI) was calculated as follows: ((number of larvae on the 24–26°C side)–(number of larvae on the 30–32°C side))/total number of larvae. Larvae that crawled off the gel or stayed at the release zone were not counted.

### Larvae optogenetic assay

The light source for the optogenetic assays consisted of a triple red (627 nm) LED starboard (07007-PD000-F, LEDSupply) mounted on a star-shaped heat sink (882-100AB, Wakefield-Vette) with thermal adhesive tape (LXT-T-12, Luxeon Star). A triple secondary optic (10507, LEDSupply) was mounted on the LED starboard with liquid adhesive (46040, Loctite). Power was supplied with a 1000 mA LED driver (3021-D-E-1000, Luxeon Star). Assays were recorded on a Sony HDR-CX405 camcorder (the internal infrared filter was removed and an 830 nm long-pass filter (FSQ-RG830, Newport) was installed) and saved as MTS files. The light source was placed under an inverted 1L beaker. A 100 mm petri dish (351029, Corning) with 20 mL of 3% agar was placed on top of the beaker and an infrared light (4331910725, Amazon) illuminated the setup to visualize the larvae.

Larvae were collected and prepared for the assay as detailed above except that they were kept in food with 40 μM all *trans*-retinal (all *trans*-retinal (ATR, Sigma-Aldrich) was dissolved in EtOH as a 40 mM stock solution) in dark for two days. Individual larvae were placed on the agar gel and given 30 seconds to acclimate. The lid was placed on the Petri dish during assays. Optogenetic stimulation was administered in 10 cycles of 5 seconds on and 15 seconds off. In **S3 Fig**, larvae avoidance behavior was manually evaluated as follows: (number of avoidant response/10 cycles) x 100%. The avoidant response was scored if the larva stopped, reversed in direction, or turned its head.

To evaluate the avoidance behavior by an automated method (**Fig 2G**), the MTS files were converted to uncompressed AVI files using the ffmpeg command line tool. The resulting files were reduced in size to 720 X 576 resolution using Any Video Converter 9 (Anvsoft). The videos were then processed using ImageJ as described with modifications [36]. The area of interest was selected using the rectangular selection tool (Image > Crop). Then, the subtract background function was used to remove continuous backgrounds from all frames (Process > Subtract background; rolling ball radius of 1; box corresponding to “light background” was selected). Next, the stack was converted to 8-bit grayscale (Image > Type > 8-bit) and the brightness and contrast were adjusted to enhance the difference between larvae and background (Image > Adjust > Brightness/Contrast). Lastly, the threshold was edited to remove the excess background so that the larvae appeared dark on a light background (Image > Adjust > Threshold. The method was set to default, the background was set to dark, and the threshold for each image was calculated).

The ImageJ plugin TrackMate was used to calculate the speed of the larvae from the larval trajectory. To locate and track the larvae, the LoG detector was used with a threshold set to 9–12 pixels and an estimated blob diameter of 9–11 pixels for 72 hr AEL larvae and 11–15 pixels for 120 hr AEL larvae. The variations were due to the difference in the distance of the camera from the Petri dish, size of larvae, and quality of the video. The HyperStack Displayer was used to visualize the regions of interest and filter out the background by setting the quality, contrast, x, and y locations for the spots tracked. The simple LAP tracker with a linking max distance of 15–25 pixels, gap-closing max distance of 15–25 pixels, and a gap-closing max frame gap between 500–1000 frames were used to generate the trajectory. The x and y position for each frame was then exported by selecting the correct tracks that represented the larvae.

The movement from one frame to another was calculated through the following formula where n is the value for the next available timepoint:

$$\Delta Distance=\sqrt{{\left(x_{n+1}-x_{n}\right)}^{2}+{\left(y_{n+1}-y_{n}\right)}^{2}}$$

TrackMate could not detect the larva when it moved into an area with the light source, the reflection of the light, or along the edge of the Petri dish. Thus, not all frames had values and the distance was calculated from the next available frame that had a tracking value. A threshold value of 2 pixels per 0.04s (one frame apart) for distance was set, and larger values were removed since these were due to artifacts such as shaking of the video or movement of the tracker position along the larval body. Trials with missing data for more than 4 flash periods were discarded. The larval speed was computed by adding the change in distance for each period (30 seconds for the control period before light stimulation and 5 seconds for the light-on period) and dividing by the time of the period. The relative speed was defined as the speed during the light-on period divided by the speed during the control period.

### Calcium imaging

Larvae were immobilized between a glass slide and a glass coverslip (22 x 40 mm) with 1x PBS. A type-N thermocouple microprobe (IT-24P, Physitemp) was also mounted between the slide and coverslip near the larvae. Imaging was performed on a Zeiss LSM 880 with Airyscan Fast mode and Definite Focus.2 to correct for focus drift due to thermal expansion and contraction. Z-stacks were acquired at 11 fps, 760x760 resolution, and 1.5 zoom using a 25x water objective. To increase the speed of z-stack acquisition, a z-axis piezo stage (432339-9000-000, Wienecke & Sinske) with stage insert (432339-9030-000, Wienecke & Sinske) was utilized.

A custom-built thermoelectric cooler was made to decrease the temperature by attaching a thermoelectric module (30 x 30 mm, TE-127-1.0–0.8, TE Technology) to a heat sink (12.9 x 5.5 cm, modified from ATS2193-ND, Digi-Key). The thermoelectric cooler was placed on the slide covering the larvae, and a 2A current was applied with a power supply (CSI1802X, Circuit Specialists). The temperature range of the thermoelectric cooler was typically 26 to 14°C. The temperature was monitored using a data acquisition device (USB-TEMP, Measurement Computing) and DAQami software (Measurement Computing). The temperature was maintained at 26°C for 30 seconds. Then, the temperature was decreased to 14°C for 30 seconds and then increased back to 26°C for 30 seconds for 3 cycles. Images were analyzed using Zeiss ZEN software. Ellipse ROIs were drawn around each neuron of interest to determine average pixel intensity. Background levels were determined by using an ellipse ROI nearby the neurons of interest. ΔF/F was calculated as follows: (F_n_−F_0_)/F_0_ x 100%.

### Immunostaining

Immunostaining was performed as described [37]. The following antibodies were used: guinea pig anti-IR21a [19] (1:100), guinea pig anti-IR93a [18] (1:100), rabbit anti-IR25a [26] (1:100), chicken anti-GFP (1:500; Abcam), goat anti-guinea pig Cy3 (1:100; Jackson ImmunoResearch), goat anti-rabbit Cy3 (1:100; Jackson ImmunoResearch), goat anti-rabbit FITC (1:100; Jackson ImmunoResearch), goat anti-chicken FITC (1:500; Invitrogene).

To quantify immunostaining, the center of each soma was determined by NIS Elements Viewer and outlined based on GFP signals (**Fig 4**) or IR21a signals (**S8 Fig**). The mean intensity was quantified using ImageJ (Analyze>Measure) and subtracted by the background intensity. The normalized fluorescence was quantified as the fluorescent intensity was divided by the average intensity of the corresponding fluorescent intensity in *wild type* animals at 72 hr AEL.

### Statistical analysis

Statistical details of experiments are mentioned in the figure legends. The normality of distributions was assessed by the Shapiro-Wilk W test (*p* ≤ 0.05 rejected normal distribution). Statistical comparisons of normally distributed data were performed by the two-tailed unpaired *t-test* or, for multiple comparisons, the Tukey test. For data that did not conform to a normal distribution, statistical comparisons were performed by the Mann-Whitney test or, for multiple comparisons, the Dunn’s test. Data analysis was performed using GraphPad Prism 8. Normally distributed data were plotted by scatterplots superimposed with bars that represent mean ± s.e.m. For data that did not conform to a normal distribution, box plots were used. In box plots, boxes are defined by 25th to 75th percentiles; internal lines show median; whiskers extend 1.5 times interquartile range; black dots denote outliers.

## Supporting information

S1 Fig Apparatus of the thermal preference assay. S1A Fig Apparatus of a temperature gradient used for testing thermal preference. One side of the aluminum plate is placed on ice, and the other side is placed on a hot plate with a set temperature. A 3% agar gel is placed in the middle as the testing surface with a temperature range from 13°C to 31°C. The measurements are in centimeters (cm). S1B Fig Actual temperatures measured at indicated gradient positions. Data represent mean ± s.e.m; n = 9.
(TIF)

S2 Fig The role of DOCCs in thermal preference at 72 hr AEL. S2A Fig Larvae distribution along a thermal gradient of indicated genotypes and ages. Data represent mean ± s.e.m; *wild type* at 120 hr AEL: n = 9; *Ir21a>TNT* (*Ir21a-Gal4/UAS-TNT*) at 72 hr AEL: n = 8. The same data from **Fig 2C and 2D**. S2B Fig Fraction of larvae of indicated genotypes and ages in the 13–21°C region. Welch’s test, F = 1.899, *p* = 0.3881.
(TIF)

S3 Fig Red-light avoidance rate of 72 hr (S3A Fig) and 120 hr (S3B Fig) AEL larvae when DOCCs express *CsChrimson* with or without dietary retinal (ATR). The genotype of *Ir21a>CsChrimson* is *Ir21a-Gal4;UAS-CsChrimson*. n = 30 except n = 29 for *Ir21a-Gal4* at 120 hr AEL with ATR. Kruskal-Wallis test. * *p* < 0.0001, Dunn’s test. Behavioral recordings in **Fig 2G** were reused.
(TIF)

S4 Fig DOCCs’ response to prolonged cool temperatures. Fluorescence change in *Ir21a-Gal4;UAS-GCaMP6m*-labeled DOCCs at 72 hr AEL is quantified as the percent change in fluorescence intensity compared to initial intensity. n = 7 cells from 3 animals. Traces, mean ± s.e.m.
(TIF)

S5 Fig The time to pupation was indistinguishable between *wild type* and *Ir* mutants, including *Ir21a*^*Δ1*^, *Ir93a*^*MI*^, and *Ir25a*^*2*^. Fraction of pupae of indicated genotypes over time. Data represent mean ± s.e.m. *wild type*: n = 9; *Ir21a*^*Δ1*^: n = 4; *Ir93a*^*MI*^: n = 11; *Ir25a*^*2*^: n = 9. Kruskal-Wallis test. Day 4: *p* = 0.8766; day 5: *p* = 0.0902; day 6: *p* = 0.4817; day 7: *p* = 0.4478; day 8: *p* = 0.9477; day 9: *p* = 0.2589.
(TIF)

S6 Fig *Ir21a*^*Δ1*^, *Ir93a*^*MI*^, and *Ir25a*^*2*^ had no general impairment in temperature discrimination or locomotion activity. Two-choice thermotactic behavioral assay was used. Larvae at 72 hr AEL were given 2 min to choose between 24–26°C and 30–32°C regions. Preference index (PI) was calculated. n = 9. Kruskal-Wallis test. *wild type* vs *Ir21a*^*Δ1*^: *p* = 0.5442; *wild type* vs *Ir93a*^*MI*^: *p* > 0.9999; *wild type* vs *Ir25a*^*2*^: *p* = 0.5191.
(TIF)

S7 Fig The role of IR21a, IR93a, and IR25a in thermal preference at 72 hr AEL. S7A, S7C, and S7E Fig Larvae distribution along a thermal gradient of indicated genotypes and ages. Data represent mean ± s.e.m; n = 9, except n = 14 for *Ir25a*^*2*^ at 72 hr AEL. The same data from **Fig 5A, 5C, 5E, 5G, 5I, and 5K**. S7B, S7D, and S7F Fig Fraction of larvae of indicated genotypes and ages in the 13–21°C region. Welch’s test. (S7B Fig) F = 1.444, *p* = 0.6157. (S7D Fig) F = 2.499, *p* = 0.2778. (S7F Fig) F = 2.262, *p* = 0.1839.
(TIF)

S8 Fig Expression of IR21a, IR93a, and IR25a in DOCCs at the late third instar is increased in *Ir21a>IR21a/93a/25a* animals. S8A, S8B, and S8C Fig IR25a immunostaining in *wild type* at 72 hr AEL (S8A Fig), *wild type* at 120 hr AEL (S8B Fig), and *Ir21a>IR21a/93a/25a* at 120 hr AEL (S8C Fig). S8D, S8E, and S8F Fig IR21a (magenta) and IR93a (green) immunostaining in *wild type* at 72 hr AEL (S8D Fig), *wild type* at 120 hr AEL (S8E Fig), and *Ir21a>IR21a/93a/25a* at 120 hr AEL (S8F Fig). Yellow arrows denote cell bodies and white arrowheads denote “dendrite bulbs.” The genotype of *Ir21a>IR21a/93a/25a* is *Ir21a-Gal4/UAS-IR25a;UAS-IR21a/UAS-IR93a*. Scale bars, 10 μm. S8G and S8H Fig Normalized somal fluorescent intensity of IR21a (S8G Fig) and IR93a (S8H Fig) at 120 hr AEL in *wild type* and *Ir21a>IR21a/93a/25a*. Mann-Whitney test, * *p* < 0.0001. IR21a: n = 109 cells from 21 *wild type* animals and n = 40 cells from 9 *Ir21a>IR21a/93a/25a* animals. IR93a: n = 90 cells from 16 *wild type* animals and n = 40 cells from 9 *Ir21a>IR21a/93a/25a* animals. The *wild type* data were the same data from **Fig 4B and 4D**. Since it was difficult to combine *UAS-GFP* with *Ir21a-Gal4/UAS-IR25a;UAS-IR21a/UAS-IR93a*, the cell bodies of DOCCs could not be precisely identified in IR25a staining and thus the quantification was not performed.
(TIF)

S9 Fig Schematic of how Rh5/6 pathway and IR21a/93a/25a pathway function in the transition of thermopreference in *Drosophila* larvae. Briefly, Rh5/6 pathway is expressed in the body wall, functions at the late third instar, and navigates animals to 18°C. However, it is unclear whether this pathway functions in driving attraction to 18°C or aversion to 24°C. IR21a, IR93a, and IR25a are expressed in DOCCs at the early third instar and drive 18°C avoidance. At the late third instar, expression of IR21a, IR93a and IR25a is decreased and thus insufficient to drive aversion to 18°C.
(TIF)

S1 Movie Red-light responses of 72 hr and 120 hr AEL larvae when DOCCs express *CsChrimson* with or without dietary retinal (ATR). The genotype of *Ir21a>CsChrimson* is *Ir21a-Gal4;UAS-CsChrimson*.
(MP4)

S2 Movie Temperature responses of DOCCs in 72 hr and 120 hr AEL larvae. The genotype of *Ir21a>GCaMP6m* is *Ir21a-Gal4;UAS-GCaMP6m*.
(MP4)
