# Correction: Ionotropic Receptor-dependent cool cells control the transition of temperature preference in *Drosophila* larvae

**DOI:** 10.1371/journal.pgen.1009631

**Published:** 2021-06-14

**Authors:** The PLOS Genetics Staff

In the Immunostaining subsection of the Materials and methods, there are errors in the second sentence of the first paragraph. The publisher apologizes for the errors. The correct sentence is:

The following antibodies were used: guinea pig anti-IR21a [19] (1:100), rabbit anti-IR93a [18] (1:100), guinea pig anti-IR25a [26] (1:100), chicken anti-GFP (1:500; Abcam), goat anti-guinea pig Cy3 (1:100; Jackson ImmunoResearch), goat anti-rabbit Cy3 (1:100; Jackson ImmunoResearch), goat anti-rabbit FITC (1:100; Jackson ImmunoResearch), goat anti-chicken FITC (1:500; Invitrogene).
