# Supplementary figures and images for: Ionotropic Receptor-dependent cool cells control the transition of temperature preference in Drosophila larvae
Source: PLoS Genet. 2021 Apr 7;17(4):e1009499. doi: 10.1371/journal.pgen.1009499 (PMC8055001; doi:10.1371/journal.pgen.1009499)

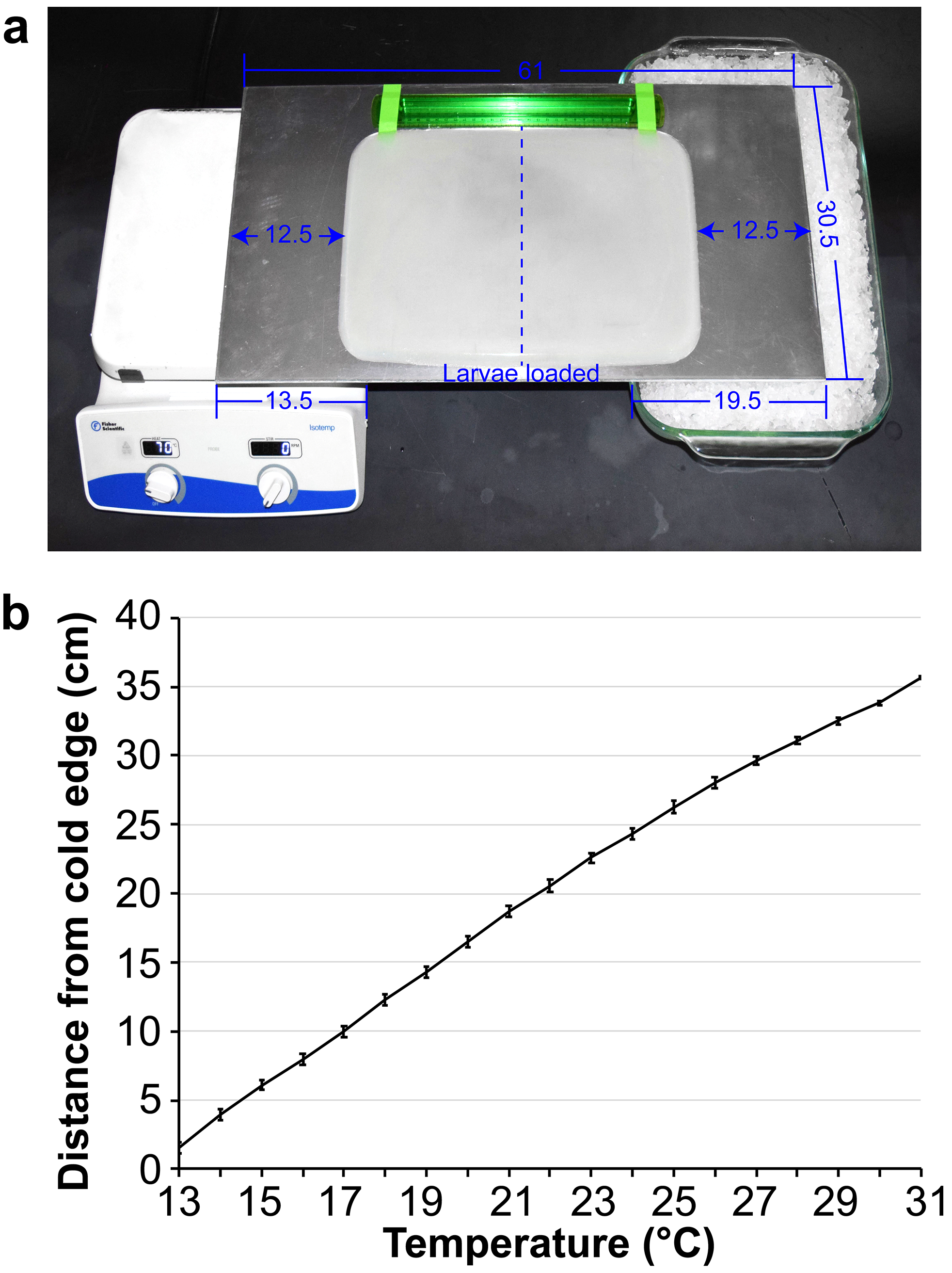

Supplement: S1 Fig — S1A Fig Apparatus of a temperature gradient used for testing thermal preference. One side of the aluminum plate is placed on ice, and the other side is placed on a hot plate with a set temperature. A 3% agar gel is placed in the middle as the testing surface with a temperature range from 13°C to 31°C. The measurements are in centimeters (cm). S1B Fig Actual temperatures measured at indicated gradient positions. Data represent mean ± s.e.m; n = 9. (TIF) [file pgen.1009499.s001.tif]

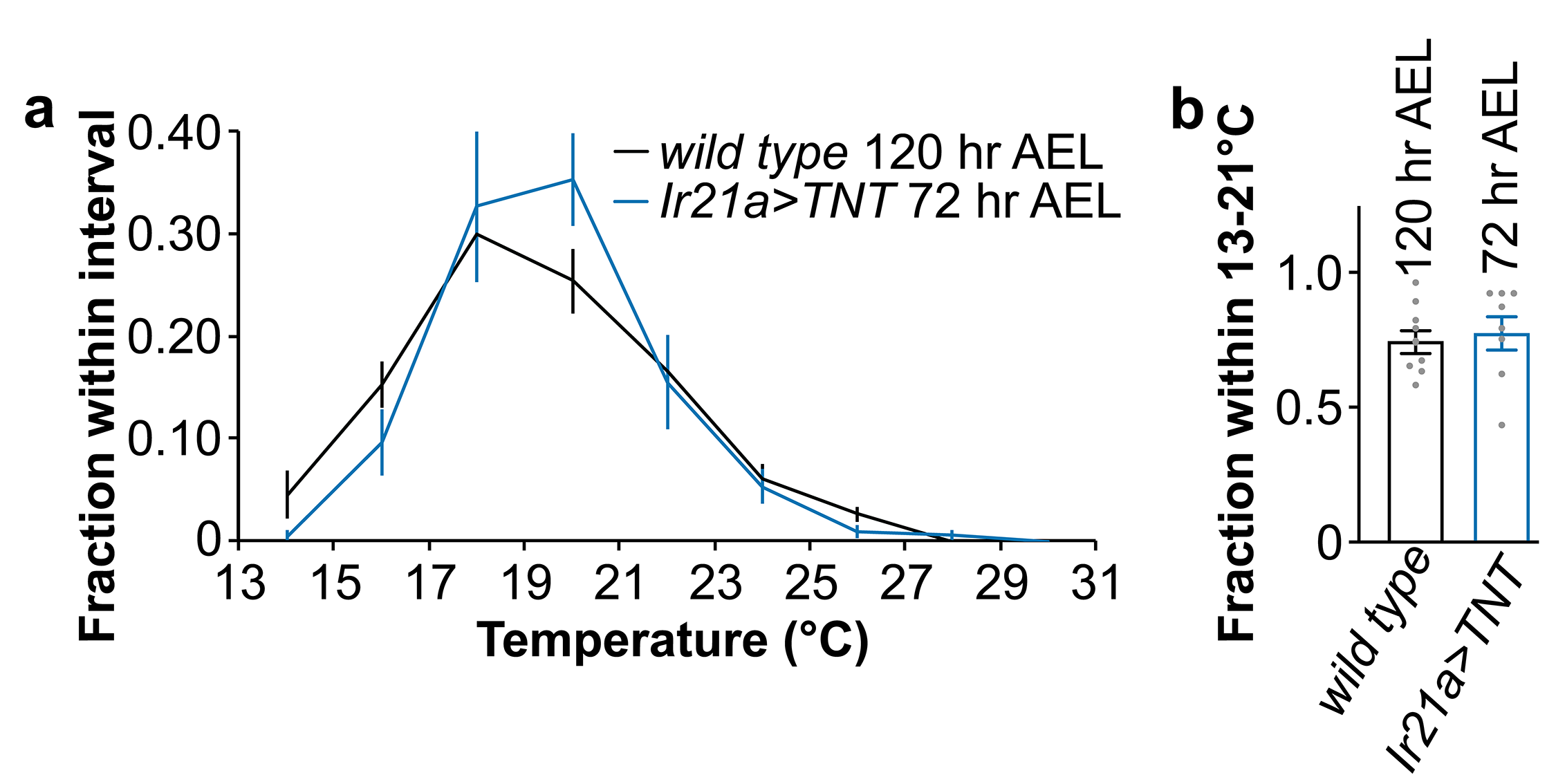

Supplement: S2 Fig — S2A Fig Larvae distribution along a thermal gradient of indicated genotypes and ages. Data represent mean ± s.e.m; wild type at 120 hr AEL: n = 9; Ir21a>TNT (Ir21a-Gal4/UAS-TNT) at 72 hr AEL: n = 8. The same data from Fig 2C and 2D. S2B Fig Fraction of larvae of indicated genotypes and ages in the 13–21°C region. Welch’s test, F = 1.899, p = 0.3881. (TIF) [file pgen.1009499.s002.tif]

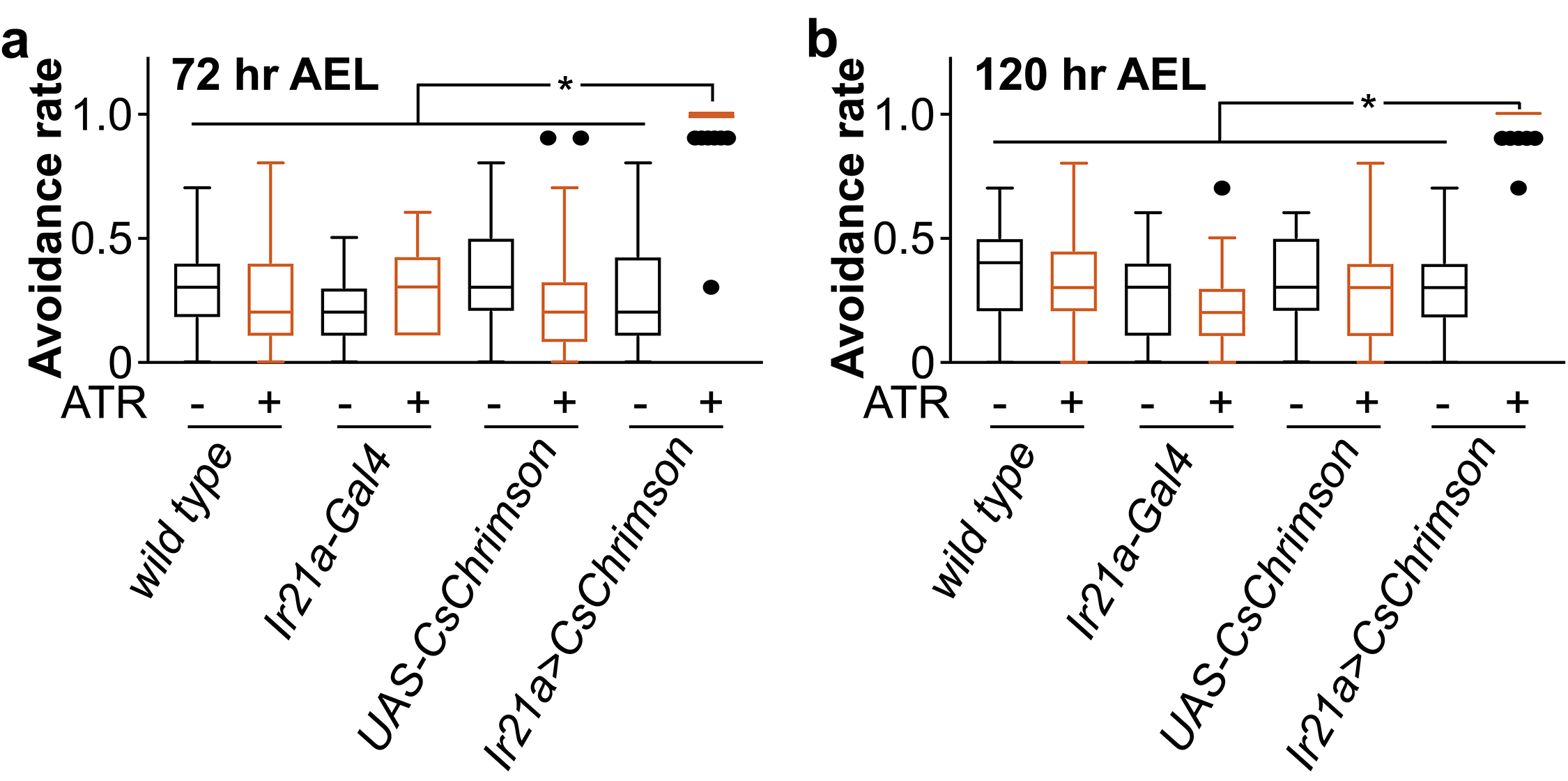

Supplement: S3 Fig — The genotype of Ir21a>CsChrimson is Ir21a-Gal4;UAS-CsChrimson. n = 30 except n = 29 for Ir21a-Gal4 at 120 hr AEL with ATR. Kruskal-Wallis test. * p < 0.0001, Dunn’s test. Behavioral recordings in Fig 2G were reused. (TIF) [file pgen.1009499.s003.tif]

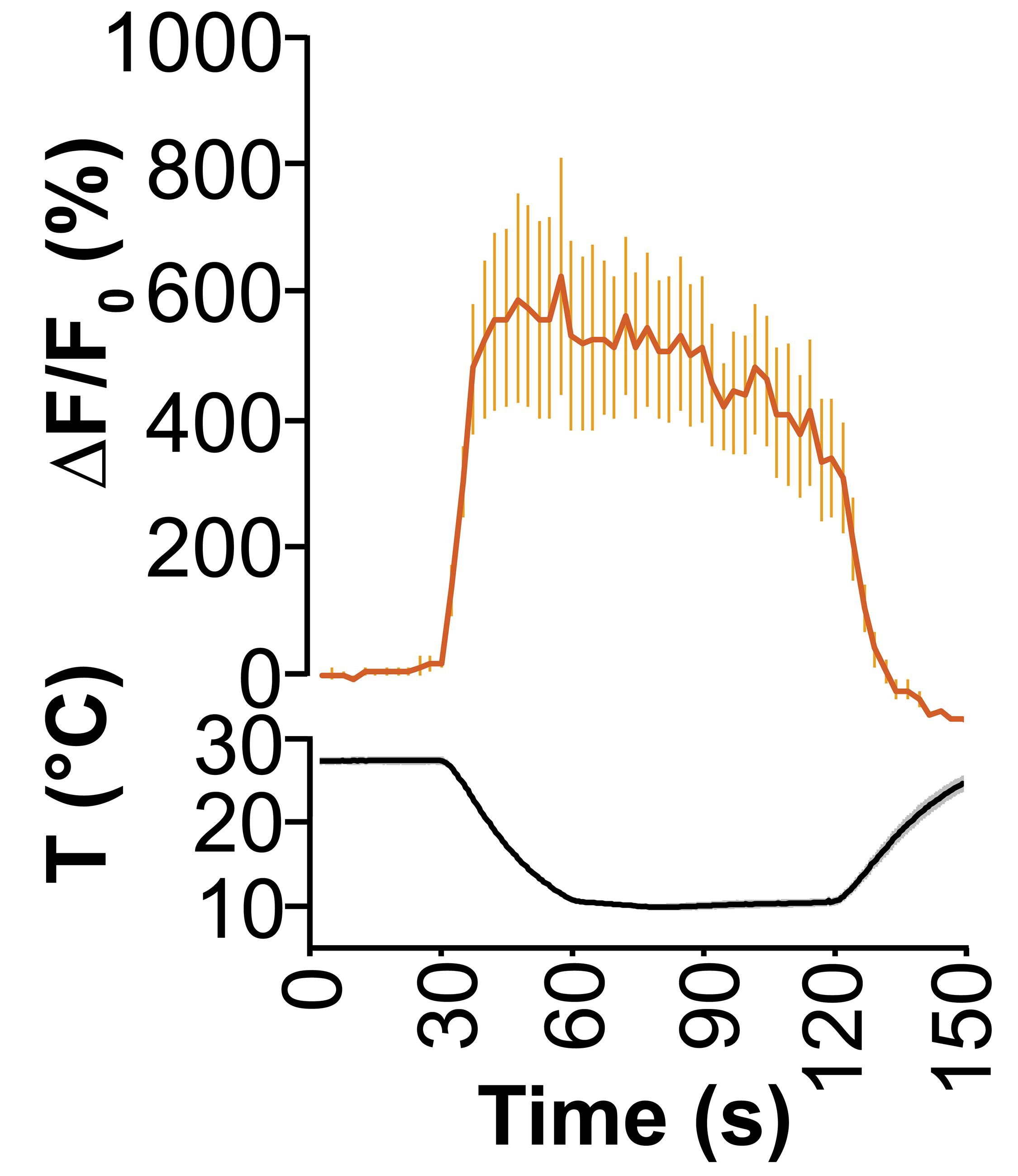

Supplement: S4 Fig — Fluorescence change in Ir21a-Gal4;UAS-GCaMP6m-labeled DOCCs at 72 hr AEL is quantified as the percent change in fluorescence intensity compared to initial intensity. n = 7 cells from 3 animals. Traces, mean ± s.e.m. (TIF) [file pgen.1009499.s004.tif]

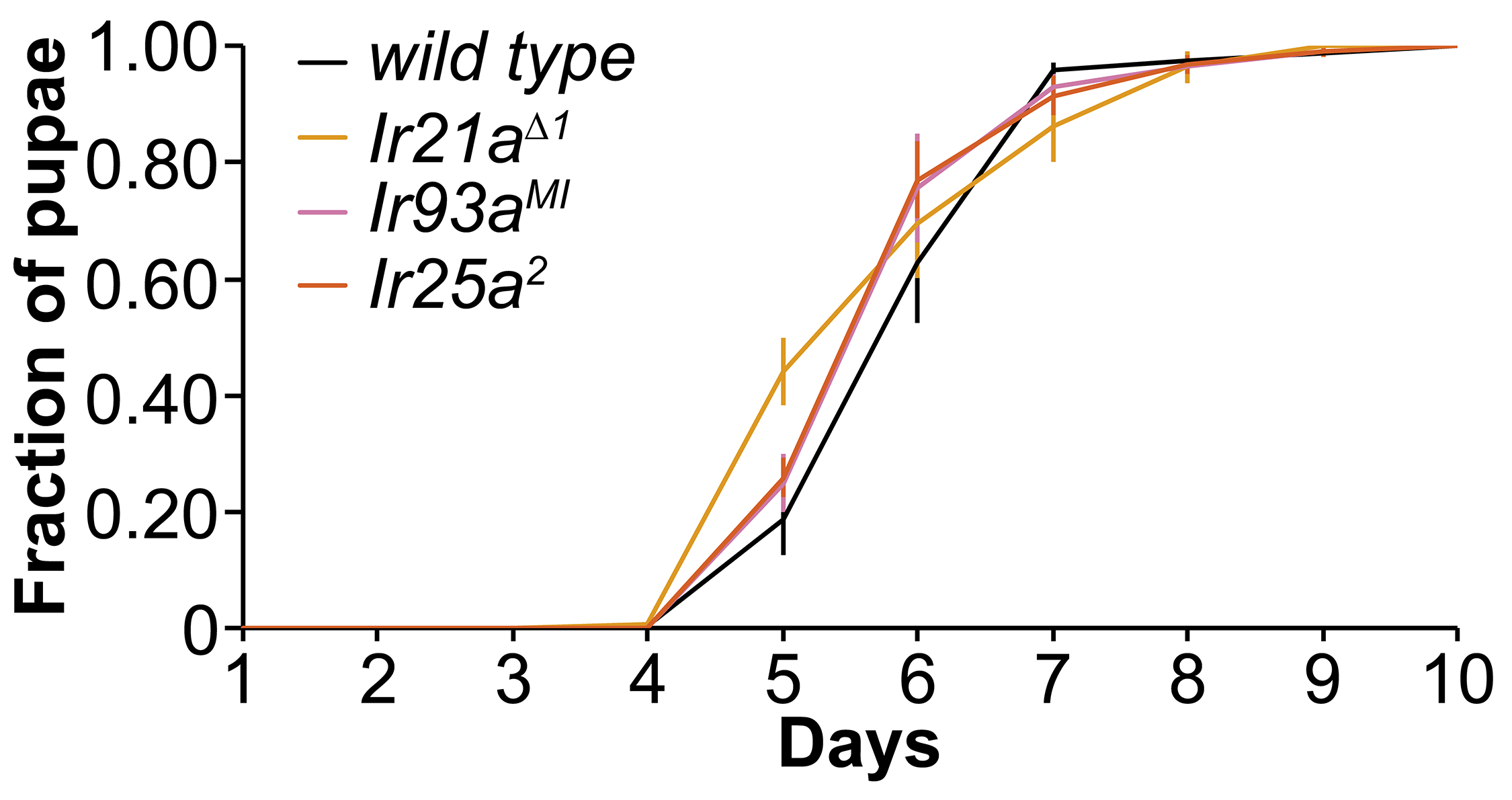

Supplement: S5 Fig — Fraction of pupae of indicated genotypes over time. Data represent mean ± s.e.m. wild type: n = 9; Ir21aΔ1: n = 4; Ir93aMI: n = 11; Ir25a2: n = 9. Kruskal-Wallis test. Day 4: p = 0.8766; day 5: p = 0.0902; day 6: p = 0.4817; day 7: p = 0.4478; day 8: p = 0.9477; day 9: p = 0.2589. (TIF) [file pgen.1009499.s005.tif]

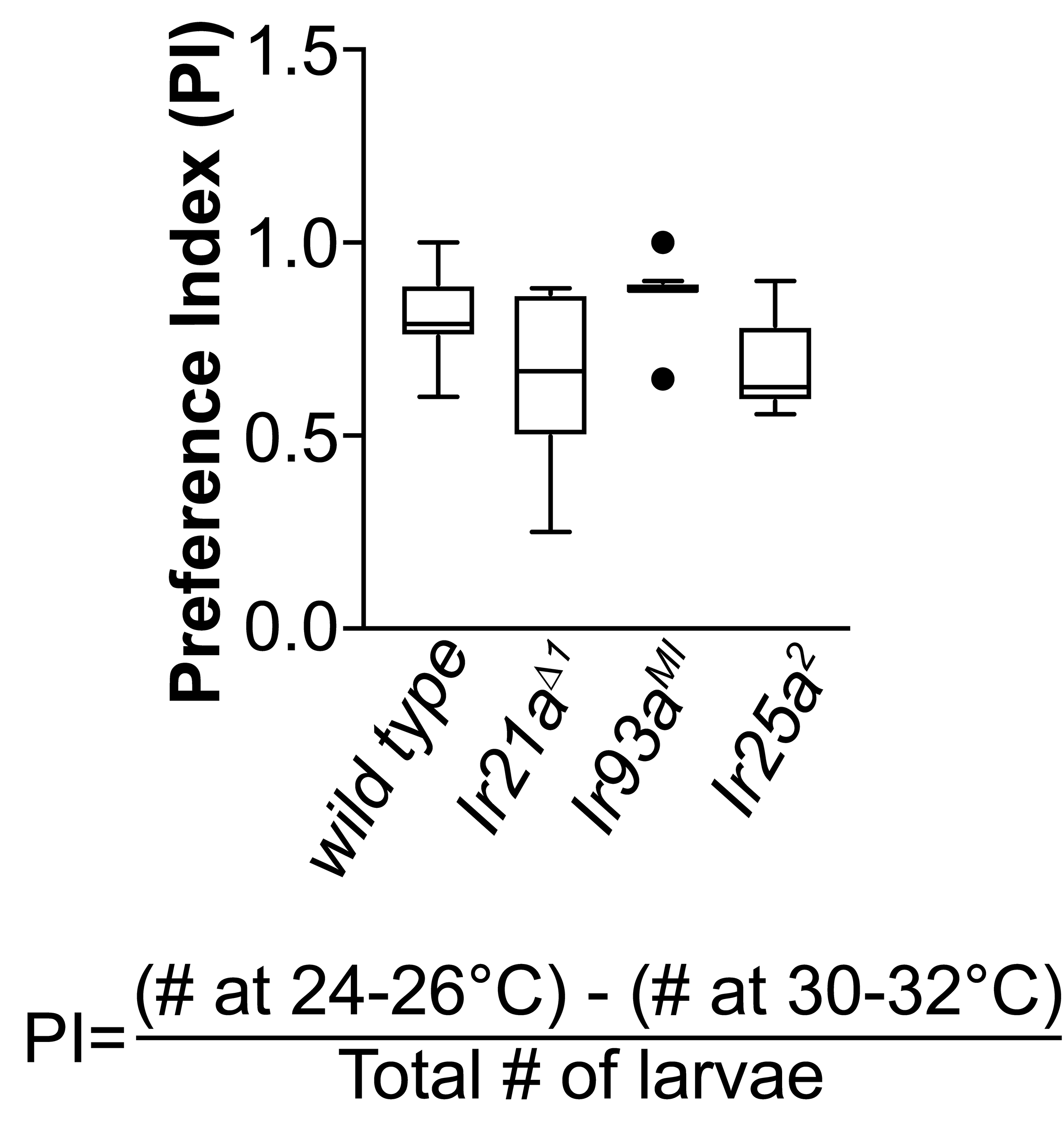

Supplement: S6 Fig — Two-choice thermotactic behavioral assay was used. Larvae at 72 hr AEL were given 2 min to choose between 24–26°C and 30–32°C regions. Preference index (PI) was calculated. n = 9. Kruskal-Wallis test. wild type vs Ir21aΔ1: p = 0.5442; wild type vs Ir93aMI: p > 0.9999; wild type vs Ir25a2: p = 0.5191. (TIF) [file pgen.1009499.s006.tif]

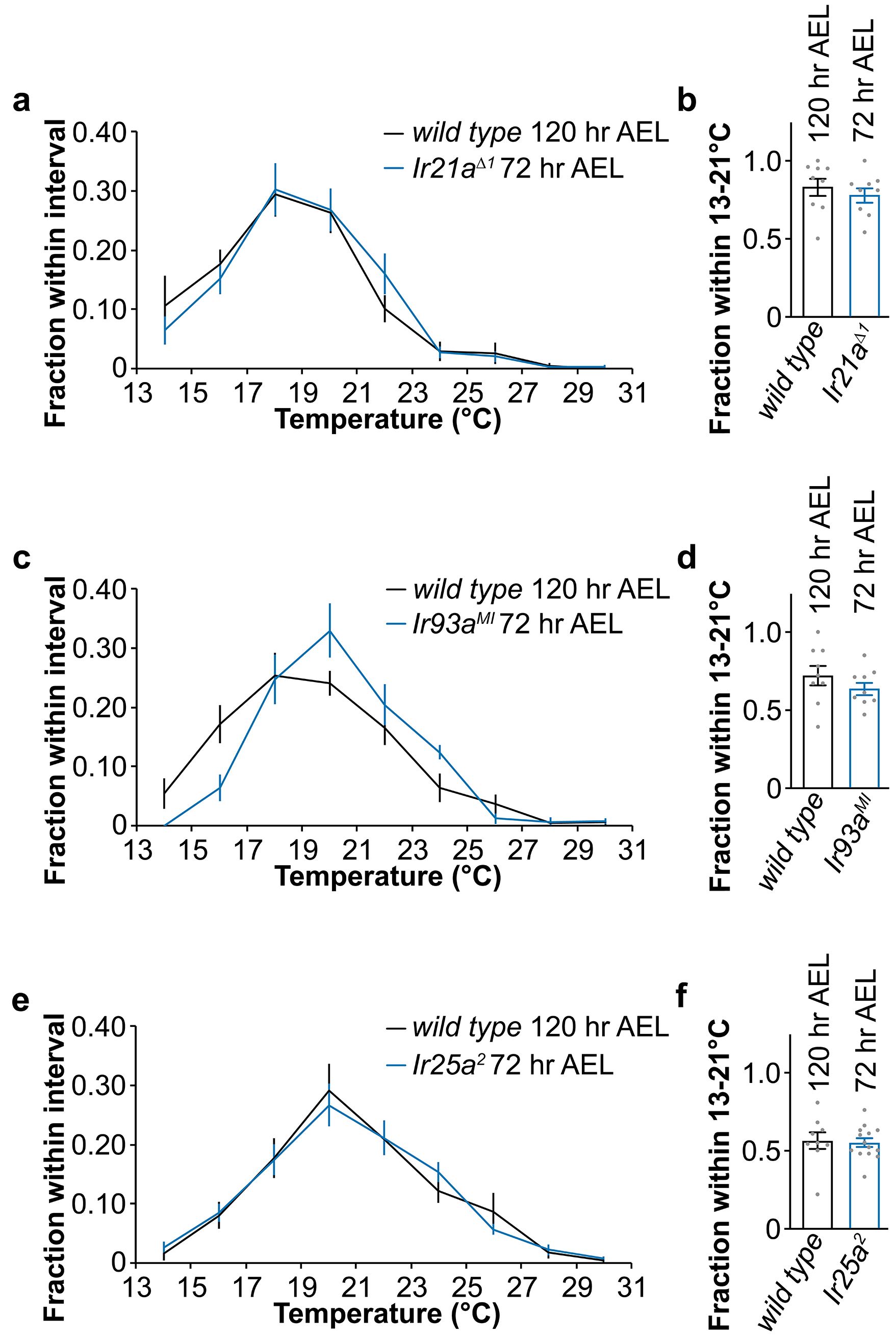

Supplement: S7 Fig — S7A, S7C, and S7E Fig Larvae distribution along a thermal gradient of indicated genotypes and ages. Data represent mean ± s.e.m; n = 9, except n = 14 for Ir25a2 at 72 hr AEL. The same data from Fig 5A, 5C, 5E, 5G, 5I, and 5K. S7B, S7D, and S7F Fig Fraction of larvae of indicated genotypes and ages in the 13–21°C region. Welch’s test. (S7B Fig) F = 1.444, p = 0.6157. (S7D Fig) F = 2.499, p = 0.2778. (S7F Fig) F = 2.262, p = 0.1839. (TIF) [file pgen.1009499.s007.tif]

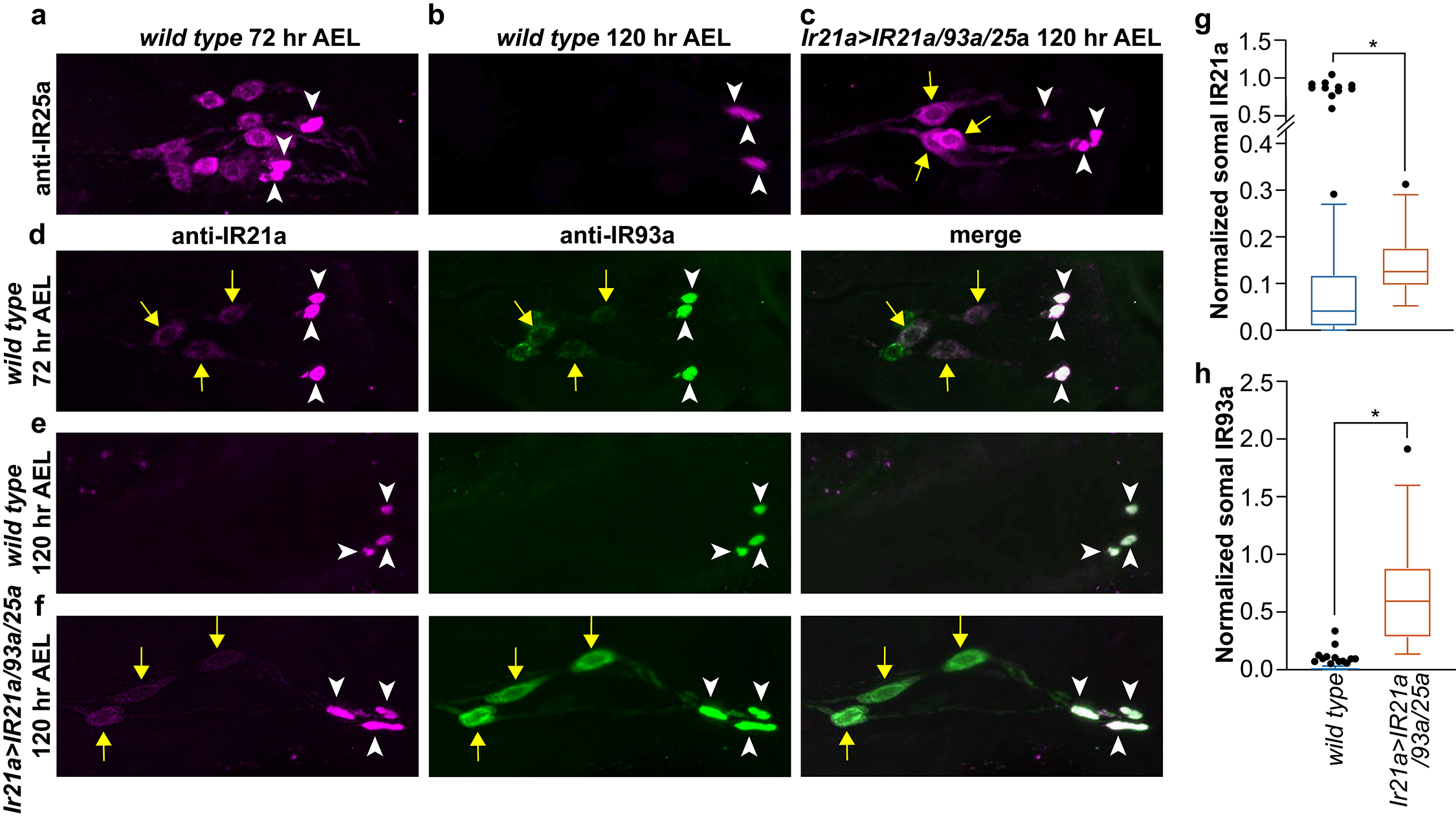

Supplement: S8 Fig — S8A, S8B, and S8C Fig IR25a immunostaining in wild type at 72 hr AEL (S8A Fig), wild type at 120 hr AEL (S8B Fig), and Ir21a>IR21a/93a/25a at 120 hr AEL (S8C Fig). S8D, S8E, and S8F Fig IR21a (magenta) and IR93a (green) immunostaining in wild type at 72 hr AEL (S8D Fig), wild type at 120 hr AEL (S8E Fig), and Ir21a>IR21a/93a/25a at 120 hr AEL (S8F Fig). Yellow arrows denote cell bodies and white arrowheads denote “dendrite bulbs.” The genotype of Ir21a>IR21a/93a/25a is Ir21a-Gal4/UAS-IR25a;UAS-IR21a/UAS-IR93a. Scale bars, 10 μm. S8G and S8H Fig Normalized somal fluorescent intensity of IR21a (S8G Fig) and IR93a (S8H Fig) at 120 hr AEL in wild type and Ir21a>IR21a/93a/25a. Mann-Whitney test, * p < 0.0001. IR21a: n = 109 cells from 21 wild type animals and n = 40 cells from 9 Ir21a>IR21a/93a/25a animals. IR93a: n = 90 cells from 16 wild type animals and n = 40 cells from 9 Ir21a>IR21a/93a/25a animals. The wild type data were the same data from Fig 4B and 4D. Since it was difficult to combine UAS-GFP with Ir21a-Gal4/UAS-IR25a;UAS-IR21a/UAS-IR93a, the cell bodies of DOCCs could not be precisely identified in IR25a staining and thus the quantification was not performed. (TIF) [file pgen.1009499.s008.tif]

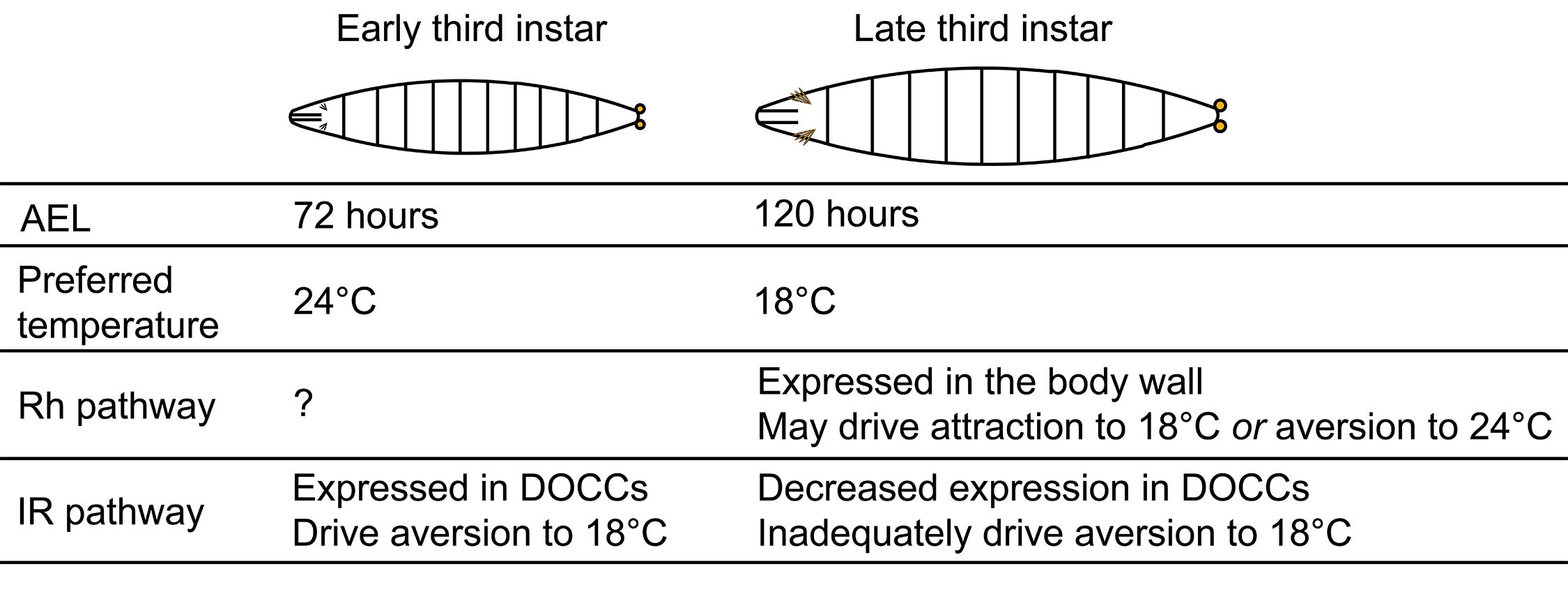

Supplement: S9 Fig — Briefly, Rh5/6 pathway is expressed in the body wall, functions at the late third instar, and navigates animals to 18°C. However, it is unclear whether this pathway functions in driving attraction to 18°C or aversion to 24°C. IR21a, IR93a, and IR25a are expressed in DOCCs at the early third instar and drive 18°C avoidance. At the late third instar, expression of IR21a, IR93a and IR25a is decreased and thus insufficient to drive aversion to 18°C. (TIF) [file pgen.1009499.s009.tif]
